# The Role of Serotonin in Fear Learning and Memory: A Systematic Review of Human Studies

**DOI:** 10.3390/brainsci13081197

**Published:** 2023-08-12

**Authors:** Francesco Tortora, Abed L. Hadipour, Simone Battaglia, Alessandra Falzone, Alessio Avenanti, Carmelo M. Vicario

**Affiliations:** 1 Dipartimento di Scienze Cognitive, Psicologiche, Pedagogiche e Degli Studi Culturali, Università Degli Studi di Messina, Via Concezione 6, 98121 Messina, Italy; francesco.tortora@uni-wuerzburg.de (F.T.); alessandra.falzone@unime.it (A.F.); 2 Centro Studi e Ricerche in Neuroscienze Cognitive, Dipartimento di Psicologia “Renzo Canestrari”, Campus di Cesena, Alma Mater Studiorum Università di Bologna, Viale Rasi e Spinelli 176, 47521 Cesena, Italy; simone.battaglia@unibo.it; 3 Centro de Investigación en Neuropsicología y Neurociencias Cognitivas, Universidad Católica Del Maule, Talca 3460000, Chile

**Keywords:** human fear conditioning, fear memory, serotonin, 5-HT receptor, selective serotonin reuptake inhibitors (SSRIs), acute tryptophan depletion (ATD), 5-HTT polymorphisms

## Abstract

Fear is characterized by distinct behavioral and physiological responses that are essential for the survival of the human species. Fear conditioning (FC) serves as a valuable model for studying the acquisition, extinction, and expression of fear. The serotonin (5-hydroxytryptamine, 5-HT) system is known to play a significant role in emotional and motivational aspects of human behavior, including fear learning and expression. Accumulating evidence from both animal and human studies suggests that brain regions involved in FC, such as the amygdala, hippocampus, and prefrontal cortex, possess a high density of 5-HT receptors, implicating the crucial involvement of serotonin in aversive learning. Additionally, studies exploring serotonin gene polymorphisms have indicated their potential influence on FC. Therefore, the objective of this work was to review the existing evidence linking 5-HT with fear learning and memory in humans. Through a comprehensive screening of the PubMed and Web of Science databases, 29 relevant studies were included in the final review. These studies investigated the relationship between serotonin and fear learning using drug manipulations or by studying 5-HT-related gene polymorphisms. The results suggest that elevated levels of 5-HT enhance aversive learning, indicating that the modulation of serotonin 5-HT2A receptors regulates the expression of fear responses in humans. Understanding the role of this neurochemical messenger in associative aversive learning can provide insights into psychiatric disorders such as anxiety and post-traumatic stress disorder (PTSD), among others.

## 1. Introduction 

Serotonin (also known as 5-hydroxytryptamine, or 5-HT) is a monoamine neurotransmitter that plays a crucial role in various physiological and pathological processes in the brain. It is an ancient chemical that existed in plants before the emergence of animal species, underscoring its fundamental importance in biological system evolution [1]. Tryptophan, the precursor of 5-HT, is an essential amino acid that is considered an integral part of the functioning and evolution of both vertebrate and invertebrate species [2,3,4]. While 5-HT is often referred to as the “happiness hormone” due to the positive effects of selective serotonin reuptake inhibitors (SSRIs) on some depressive disorders, its functions extend far beyond mood regulation. 5-HT is involved in the regulation of anxiety, mood, learning, memory, sleep, circadian rhythms, motor activity, locomotion, social interactions, social status, aggressiveness, and more [3]. Scholars propose brain 5-HT has two key functions: moderating anxiety and stress while promoting patience and coping [5,6], as well as facilitating adaptive psychophysiological reactions to threatening situations through plasticity [7]. 5-HT is involved in both “passive coping” (tolerating psychological pain) and “active coping” (actively dealing with psychological pain by changing one’s relationship with it) [8]. Additionally, 5-HT has been implicated in aggressive behaviors and in the encoding of social status in animals [9,10,11]. For instance, a seminal study in crayfish showed that the amount of 5-HT in the nervous system is modulated after fights [12]. This study found that the level of expression of the 5-HT receptors reflects the dominance of the animal involved in the battle. The winner had higher levels of serotonin and a greater probability of mating, therefore passing certain serotonin-related mutations [9,11]. It has also been suggested that 5-HT acts on risk perception, affecting the decision-making process related to fight or flight behavior [13,14]. This, in turn, affects fear learning and memory, which are crucial to survival because they contribute to adaptive reactions to threatening situations [15,16,17]. 

From an evolutionary perspective, it is highly advantageous to retain vivid memories of significant life experiences to learn from mistakes and avoid potential dangers in the future [18,19,20]. However, when fear learning and fear memories become abnormal and persistent, they can contribute to mental disorders like anxiety or post-traumatic stress disorder (PTSD) [21,22,23,24,25,26,27,28,29]. 5-HT has been implicated in the development and maintenance of these disorders, as well as depression [30,31,32]. Dysfunction in the serotonergic system can lead to abnormal integration of environmental information, potentially contributing to the development of these clinical conditions [33]. There is evidence suggesting that depression and anxiety are associated with alterations in associative fear learning [34,35,36,37,38]. Alterations in the 5-HT system may contribute to the pathogenesis of these clinical conditions [39], given the involvement of 5-HT in strengthening synaptic connections related to affective learning [40,41,42].

Humans are highly sensitive to potential threats [43,44,45,46,47] and can predict danger based on learned fear, an ability crucial for activating defensive behaviors and ultimately increasing the likelihood of survival [48,49,50]. 5-HT has a significant influence on affective processes [51,52,53]. Numerous studies, involving both animals and humans, have investigated the role of 5-HT in fear learning and memory using the well-established Pavlovian fear conditioning (FC) paradigm [54,55]. Indeed, FC is the most widely used experimental paradigm used for investigating the psychophysiological processes and neural mechanisms underlying the learning of potentially dangerous events [56,57]; it is also considered a useful laboratory model for understanding the neural and psychophysiological bases of psychopathologies such as anxiety disorders or PTSD [17,58,59,60,61,62,63] and studying social attitudes [64].

FC represents the process by which a stimulus comes to evoke fear after being paired with an aversive event [65]. In the FC paradigm, an initially neutral stimulus, such as a tone or an image (conditioned stimulus—CS), is presented in close temporal and contingent association with an aversive stimulus, such as a mild electric shock or unpleasant sound (unconditioned stimulus—US). Through repeated pairings, the CS acquires the property to elicit fear responses (conditioned response—CR) that were previously evoked by the US [48,57,66]. The dependent variables in FC typically include behavioral or physiological responses, such as freezing, changes in skin conductance response (SCR), startle response, and heart rate measures [57,67]. FC is widely used in several species, including humans, and there are several variants of the classical protocol [48,57]. For example, researchers have explored immersive reality versions of the Pavlovian FC protocol, leveraging the growing interest in metaverse applications in the fields of psychotherapy and rehabilitation [68]. These immersive reality versions have shown equivalent [38] or even better results [69] than standard paradigms in terms of efficiency in FC and extinction. The metaverse offers solutions that overcome the limitations of low ecological validity presented by the scenarios displayed on computer screens. It allows individuals to perceive themselves within simulated environments, facilitating experiences similar to those in reality [70,71,72,73,74,75].

Over more than a century of research utilizing the FC paradigm, our understanding of the neural bases of aversive learning has significantly advanced [76,77,78,79,80]. Modern neuroimaging techniques have expanded our knowledge of the brain networks underlying FC in humans [81]. Consistent findings across studies have demonstrated the crucial role of the amygdala in the acquisition and expression of conditioned fear, while the prefrontal cortex (PFC) and hippocampus have been implicated in various aspects of the FC process, including acquisition, storage, retrieval, expression, and contextual modulation [18,78,81,82,83]. Specifically, the acquisition of the CS-US contingency requires coordinated processes supported by an extended brain network involving cortical and subcortical areas [34,36,50,66,84,85,86,87,88]. Among these areas, the amygdala, particularly the basolateral nucleus (BLA), plays a critical role in establishing the association between the CS and US and translating them into autonomic reactions [15,16]. Furthermore, FC engages other regions such as the insula [82,89] and the anterior cingulate cortex [89], which are regions associated with emotion and defensive reactions. Recent evidence indicates that the PFC, especially the medial PFC (mPFC), is responsible for the long-term storage and retrieval of fear memories [90]. Moreover, scholars have suggested that the ventral part of the mPFC is critical for fear acquisition [76]. Lastly, the hippocampus provides contextual information related to learning, and studies have shown its crucial role in encoding and conveying contextual representations through its projections to the amygdala, which ultimately induce defensive behavior [91,92,93]. 

On the other hand, repeated presentation of the CS without the US typically leads to the gradual weakening of the CR, known as extinction [94]. During extinction, a new inhibitory association is formed, allowing the organism to discern between safe and dangerous contexts. The extinction process involves a new learning process that occurs in addition to fear acquisition [94], and it is supported by the integrated functioning of the FC network [19,56,95,96]. Research across different species has provided evidence of the phylogenetic conservation of the brain mechanisms underlying fear acquisition and extinction [19,56,95,96].

In sum, fear acquisition, memory, and extinction critically depend on the interplay between subcortical and cortical areas [97]. The amygdala plays a key role in the acquisition and expression of fear, while the PFC and hippocampus help to regulate the fear response based on contextual information, determining whether a situation is safe or dangerous [98,99,100]. The amygdala, hippocampus, and PFC contain a significant number of 5-HT receptors [68,101,102,103,104]. The amygdala, in particular, exhibits a high density of 5-HT-releasing fibers originating from the midbrain’s raphe nuclei [105]. Studies have shown that stress conditions and the presentation of US during FC increase 5-HT concentration in the amygdala [106,107,108]. Additionally, both BLA and mPFC exhibit increased extracellular 5-HT levels after contextual conditioning [106,109,110]. 

To date, numerous studies have provided evidence regarding the role of 5-HT in aversive learning, primarily based on animal models. Conducting research in humans presents challenges due to environmental and demographic factors, as well as limitations in studying brain functioning with the necessary spatial and temporal resolution [67,104]. Despite these challenges, evidence supports the importance of 5-HT in threat learning in humans as well [111]. In fact, manipulations of 5-HT levels are widely used in the treatment of anxiety disorders in humans [112,113,114,115,116]. 

It is important to note that there is a considerable amount of evidence highlighting the key role of 5-HT in human aversive learning. However, results in this field are often presented in a heterogeneous manner between animal and human experiments. Given the previous premises and the clinical impact of treatments involving modulation of the 5-HT system, it is crucial to provide an updated review of the existing literature that addresses the role of 5-HT in human fear memories.

The human 5-HT system has been extensively studied using a range of methods. These approaches encompass enhancing the 5-HT system using selective SSRIs, suppressing it through acute tryptophan depletion (ATD) and 5-HT antagonists, and investigating the impact of natural variations in 5-HT-associated genes on fear learning and memory. 

To assess the effects of 5-HT system modulations, researchers have used physiological measures reflecting autonomic nervous system activation, such as SCR, electrocardiogram (ECG), and eye blink startle response, as well as imaging techniques like functional magnetic resonance imaging (fMRI) and positron emission tomography (PET). Moreover, some studies have relied on explicit self-reports as outcome measures. 

The objective of the present systematic review was to gather and synthesize the existing literature on the involvement of 5-HT in fear learning and memory in humans. Specifically, we examined the influence of three factors: (i) SSRIs and other 5-HT modulators; (ii) Tryptophan depletion; and (iii) 5-HT-related gene polymorphisms. These factors were analyzed across 29 articles to investigate their influence on fear learning and memory. Our work provides a comprehensive qualitative overview of the evidence on the role of 5-HT in fear learning and memory in humans. We discuss the results of the review in light of the interactions between genetic factors, brain mechanisms, and behavior in the context of 5-HT and fear learning and memory. Overall, this work contributes to our understanding of the mechanisms underlying fear-related processes and offers insights into potential therapeutic interventions for fear-related disorders.

## 2. Methods

The search of relevant articles was conducted in the PubMed and Web of Science (WOS) databases on March 2021, using the following keywords: “threat conditioning”, “serotonin”, “fear”, “fear conditioning”, “fear memory”, and “5-HT”, with the additional keyword “humans” searched in All Fields. The search strategy involved considering all other keywords in the Title and Abstract searches within both PubMed and WOS. 

Inclusion criteria were defined as follows: English papers investigating fear learning and/or memory in humans using conditioning paradigms or other suitable methods to study fear-associated memory. Studies that explored the role of 5-HT through genotyping, brain imaging, 5-HT precursor manipulation (e.g., tryptophan depletion), or SSRI administration were also included. 

Two researchers independently evaluated all the selected papers to ensure they met the inclusion criteria. Papers meeting these criteria were downloaded for a comprehensive review in both databases, and further evaluation led to the exclusion of papers that did not meet the inclusion criteria. Finally, the results of independent screening and inspections of PubMed, WOS, and other sources (e.g., references) were compared and duplicates were then removed. In total, 714 studies were considered, out of which 29 were ultimately included in the final review. 

**Figure 1:**
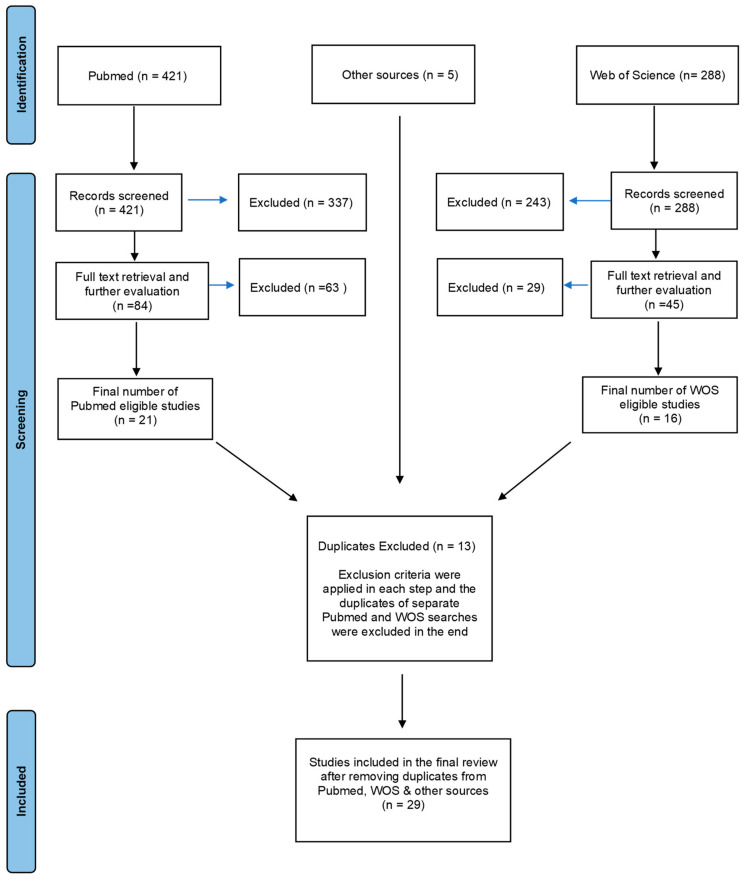
PRISMA diagram.

The exclusion criteria were as follows: animal studies, studies not written in English, review articles, and studies not using FC paradigms. Despite the limited number of studies on clinical samples (n = 2), they were not excluded and will be discussed in this review. The step-by-step outline of the search procedure is presented in Figure 1, following the format of the Preferred Reporting Items for Systematic Reviews and Meta-analyses (PRISMA) diagram [117].

The included papers are presented in two sections: (1) studies using the administration of SSRIs, other 5-HT receptor antagonists, or tryptophan depletion without investigating the potential effects of gene polymorphisms; and (2) studies specifically focusing on investigating gene polymorphisms. Within each section, the reviewed papers are presented chronologically, starting from the earliest to the most recent publications. 

## 3. Results

### 3.1. Studies on 5-HT Modulators or Tryptophan Depletion without Genotyping

The studies in this section investigate how 5-HT levels affect fear learning. We also included a study that assessed brain 5-HT levels through PET imaging, without using any of the experimental manipulations mentioned above.

#### 3.1.1. Effects of SSRIs and Other 5-HT Modulators

A pioneering study by Hensman et al. [118] examined the potential effects of ritanserin, a selective antagonist of 5-HT2 receptors in the brain, on aversive learning. The study utilized a single-trial Pavlovian FC paradigm. The participants were exposed to 10 neutral tones (CS) during the habituation phase. In the acquisition session, one CS was paired with a sudden aversive burst of white noise (US), followed by another 10 CS presentations in the extinction phase. The results indicated that participants receiving ritanserin showed reduced amplitude and spontaneous fluctuations of SCRs during the extinction phase. This suggests that reducing 5-HT levels might impair the acquisition and/or physiological expression of fear as measured by SCR and its spontaneous fluctuations [118]. 

To further investigate the role of 5-HT in human fear learning, Hetem et al. [119] administered d-fenfluramine (FEN), a drug that increases 5-HT levels by stimulating synaptic release of 5-HT and blocking its reuptake into presynaptic terminals (SSRI). The same FC paradigm used in the previous study [118] and a simulated public speaking (SPS) task were adopted to assess anxiety. The results showed that FEN administration led to decreased anxiety levels in the SPS task, in a dose-dependent manner (15 and 30 mg). However, there was no effect of the drug on SCR amplitude or fluctuations following the presentation of the CS during the extinction phase [119].

Hellewell et al. [120], using the same FC paradigm procedure as in Hensman et al. [118], investigated the effects of three different drugs on aversive conditioning. The drugs tested were buspirone (an azapirone, 5-HT1A partial agonist), fluvoxamine (an SSRI), and diazepam (a benzodiazepine). Buspirone led to faster extinction of fear responses, but it also resulted in faster SCR habituation. Similar effects were reported for fluvoxamine and diazepam. The authors proposed that such effects were attributed to the inhibitory effects that these drugs have on the dorsal raphe 5-HT2 system [120].

In another study by Silva et al. [121], the effects of the antidepressant agent nefazodone (an antagonist of 5-HT2A receptors and inhibitor of 5-HT transporter) were assessed on SCR using the FC paradigm of Hensman et al. [117]. The study found a significant reduction in spontaneous fluctuations of SCR during habituation and extinction phases, but only in the 200 mg group. This implies a reduction in arousal after drug administration, which is believed to reduce 5-HT levels in the brain. Additionally, the authors reported increased anxiety measured by an SPS task after drug administration [121]. 

Grillon et al. [122] investigated the effect of acute citalopram (an SSRI) administration on fear responses. The researchers employed a fear-potentiated startle paradigm to measure two types of threat responses: phasic cued fear and sustained startle potentiation. Phasic cued fear was assessed by comparing the startle response before and during stimulus presentation, while sustained startle potentiation was measured by comparing startle responses during inter-trial intervals of predictable and unpredictable conditions with the neutral condition. The authors implemented an instructed FC paradigm where participants were fully informed about contingencies and could observe them on a monitor during the experiment. The results indicated that prior to the initiation of conditioning, citalopram administration did not affect the amplitude of the startle. However, citalopram increased fear, potentiated startle responses to threat cues, and sustained potentiated startle responses to unpredictable shocks. Additionally, the drug increased the contextual startle potentiation in the early phase of the FC [122]. 

In a subsequent study by Grillon et al. [123], employing the same procedure as in their earlier work [122], the researchers examined the effects of administering citalopram for two weeks in healthy participants. This study revealed a significant effect on contextual anxiety (i.e., sustained startle potentiation) but not on cued fear. Interestingly, despite increased 5-HT levels in the active group, no differences in short-duration fear-potentiated startle were observed between the two groups (as measured by the difference between startle response during the presentation of the stimulus and ITI). The authors suggested that the administration of this specific SSRI for 2 weeks in healthy individuals led to a decrease in “conditioned” sustained anticipatory anxiety due to the long-duration diffused cue. This differential effect of citalopram administration was attributed to potential inherent differences between the neural mechanisms in the brain regulating the two types of learning, namely cued fear versus contextual anxiety, which would involve the central nucleus of the amygdala (CeA) and the bed nucleus of the stria terminalis (BNST), respectively [123]. 

Bui et al. [124] conducted a study to investigate the impact of escitalopram, another SSRI, on fear acquisition and extinction in healthy humans. Participants were administered either 10 mg/day of escitalopram or a placebo for two weeks. Following the treatment, a FC paradigm was carried out over two days, comprising habituation and acquisition on day 1 and extinction on day 2. Electric shocks served as the US, and SCR was used as the physiological measure. The study revealed that escitalopram had no effect on fear acquisition, but it did enhance extinction learning [124]. The absence of escitalopram effects on fear acquisition is consistent with Grillon et al. [123], who reported that citalopram had no impact on cued fear responses despite showing an anxiolytic effect on anxiety-potentiated startle. 

Åhs et al. [125] explored whether individual differences in the regulation of serotonergic transmission, measured by means of PET with [11C]-DASB, a 5-HT transporter radioligand, could predict the magnitude of fear learning in a FC task. The authors reported a negative correlation between fear learning and the binding potential of [11C]-DASB in the brain regions associated with FC. Increased absorption of this tracer is linked to lower synaptic 5-HT levels [126], and reduced systemic 5-HT levels have been found to diminish conditioning efficacy [127]. The study found negative correlations between acquisition of fear and DASB binding in the right amygdala, dorsal anterior cingulate cortex (dACC), and anterior insula, as measured by PET [125].

In a recent study, Gorka et al. [128] utilized the same FC paradigm employed by Grillon et al. [122] to compare the effects of two interventions, SSRI and cognitive behavioral therapy (CBT), in a clinical sample of patients with principal fear and distress/misery disorders. The study aimed to observe changes in startle magnitude in response to unpredictable threats. The results showed that individuals with fear-based disorders exhibited higher baseline startle magnitudes when compared to healthy controls. Interestingly, from pre- to post-treatment, patients who underwent CBT showed a reduction in startle magnitude during unpredictable threats, while those on SSRI did not exhibit the same reduction. 

Overall, these findings offer moderately consistent results on the effects of 5-HT modulators on fear expression. In studies using 5-HT antagonists, fear learning was interfered with, as these substances suppressed fear responses or facilitated the extinction process, as evident from reduced SCR levels during extinction. Conversely, studies employing different FC paradigms reported that acute administration of SSRIs and other 5-HT agonists enhanced fear learning. However, prolonged administration of these agents did not alter cued fear responses; instead, they primarily affected anxiety symptoms, leaving fear unchanged. Refer to Table 1 for a summary of studies on the influence of SSRI and other 5-HT modulating drugs on fear learning.

#### 3.1.2. Effects of Acute Tryptophan Depletion (ATD) on 5-HT Levels

Tryptophan is an essential amino acid found in various proteins in both animals and plants. Since the body cannot synthesize it, it must be acquired through the diet. Besides serving a structural role in the body and participating in protein construction, tryptophan also acts as the precursor of 5-HT [129]. The ATD technique has been employed to investigate the impact of 5-HT reduction in FC (for a review, see [130]).

In a study by Robinson et al. [131], researchers used ATD in an experimental paradigm similar to the one adopted by Grillon et al. [122] to examine its potential effects on phasic fear versus sustained anxiety. The results showed that fear-potentiated startle was not affected by ATD. However, anxiety-potentiated startle was increased by it, suggesting that the lack of 5-HT may lead to heightened anxiety, as measured by the startle response [131].

Hindi Attar et al. [127] used a standard ATD paradigm to lower the level of 5-HT in the brain. They investigated the effects of this depletion on fear learning using a Pavlovian classical conditioning paradigm in which aversive temperature changes on the skin were used as the US. The difference between predicted and actual outcomes, known as prediction error (PE), was used as a regressor in the fMRI conditioning task. The results showed that 5-HT depletion impaired fear leaning, as evidenced by decreased PE signals in the right amygdala and the left orbitofrontal cortex (OFC, involved in affective regulation). The study also found that 5-HT depletion reduced behavioral expectancy ratings and SCR levels when anticipating an aversive stimulus (i.e., being exposed to CS+ and expecting US). Overall, the study provides evidence that 5-HT plays a significant role in fear acquisition and in brain areas associated with fear learning. It suggests that a decreased level of 5-HT in the brain can directly hinder the process of fear learning [127]. 

In the most recent ATD study, Kanen et al. [132] investigated how reducing the level of 5-HT affects fear expression and extinction in a two-day FC paradigm. On the first day, all participants underwent the acquisition of FC without any manipulations in the 5-HT system, using two different CS+. Subsequently, extinction of only one of the two CS + was performed. One group of participants then underwent ATD through diet modifications, while the other group received a placebo. On the second day, the spontaneous recovery procedure was followed by a re-exposure to all CSs. Immediately after, the fear reacquisition procedure was administered in the same way as the acquisition procedure that occurred on the first day. The results showed that ATD reduced the fear response to both CS+ during spontaneous recovery. Furthermore, the higher the levels of tryptophan depletion, the lower the fear associated with SCs was. In conclusion, the authors showed how diminished levels of 5-HT blunt emotional responses to threatening stimuli during spontaneous recovery and that this effect positively correlated with uncertainty and anxiety scores as measured by the IUT survey [132].

In general, the results of studies that used ATD as an experimental manipulation appear to be moderately consistent. Both Kanen et al. [132] and Hindi Attar et al. [127] showed that 5-HT reduction blunts fear responses, even though they used different paradigms and outcome measures. Intriguingly, Robinson et al. [131] showed that 5-HT reduction does not have an effect on phasic fear but increases sustained anxiety. This result seems to be in line with evidence found by Grillon et al. [123], in which an increase in 5-HT levels caused by acute administration of citalopram reduced contextual anxiety. Substantively, both studies that have used drugs and those that have used ATD to modulate serotonin levels seem to argue in favor of a central role for 5-HT in fear learning. Broadly speaking, it appears that an increase in 5-HT levels causes an increase in physiological fear responses, while a decrease in 5-HT causes the opposite effect. However, the results in question should be interpreted with caution for different reasons. Indeed, the studies considered used different conditioning paradigms and different outcome measures that are difficult to compare. Also, some studies show effects on different physiological and behavioral components of the “fear” system (i.e., cued fear or contextual anxiety), which depend on both the manner of drug administration (chronic or acute) and the type of paradigm used. Based on these premises, it can be concluded that 5-HT is essential in fear learning processes. However, the precise mechanisms by which 5-HT influences these processes remain unclear. Refer to Table 2 for a summary of studies investigating the effect of ATD on fear learning.

### 3.2. Investigations of Genetic Modulation of 5-HT in Fear Learning and Extinction

The studies in this section investigated the impact of different gene polymorphisms related to the 5-HT transporter (5-HTT) and their interaction with other genetic variations and environmental factors on the processes underlying FC.

In a seminal study, Garpenstrand et al. [133] examined the impact of 5-HTT and monoamine oxidase type A (MAOA) gene polymorphisms on fear learning and extinction using electric shock and SCR. The authors found that differences in fear acquisition and extinction were related to MAO-A protein activities and 5-HTT genotypes. They reported that carriers of the short (S) alleles of the 5-HTT-linked promoter region (5-HTTLPR) polymorphism showed better fear acquisition but not extinction compared to carriers of the long (L) alleles. Furthermore, participants with low MAO-A activity exhibited better fear acquisition, providing further evidence for the role of 5-HT in human FC [132]. 

Another study by Lonsdorf and colleagues [134] studied the potential effects of 5-HTT gene polymorphisms involved in fear acquisition and expression. The results showed that carriers of the S allele had a greater startle in response to CS+ both during the acquisition and extinction sessions, which were 24 h apart. This evidence emphasizes the interdependence between genetic components and human behavior and may also be interpreted in light of the findings regarding the connection between the S allele and neuroticism as well as an increased risk of developing anxiety disorders [134].

In a study by Crişan et al. [135], an observational FC paradigm was used to investigate the effect of 5-HT gene polymorphism on social FC. During the paradigm, participants watched a videotape of a classical FC session without being directly exposed to the US themselves (i.e., vicarious FC). The results showed an acquisition of fear through observation, and further analysis revealed a significant effect of the S allele on the SCR levels in response to the CS+. Additionally, the Heart Rate Variability (HRV) analysis revealed a significant effect of the S allele on vagal tone, indicating less vagal tone and increased sympathetic activity compared to L-homozygotes [135]. This provides a mechanistic explanation for associations between genetic variability in 5-HT function and psychophysiological measures of emotional learning, showing increased sympathetic responses to threat in S allele carriers, reflecting modulation of fear responses, and thereby converting active defensive behavior to freezing. 

Another study by Klumpers et al. [136] found that participants with at least one S allele for the 5-HTTLPR polymorphism exhibited stronger fear-potentiated startle under threat in an instructed FC paradigm. However, the downregulation of the fear response (measured by a startle response to a delayed startle stimulus after the onset of the fear cue) was not significantly different between the carriers of the S and L alleles (not in the context of fear extinction but conceptualized as less startle response shortly after the stimulus onset) [136]. 

In their study on fear memory and reconsolidation processes, Agren et al. [137] investigated the relationship between 5-HT- and dopamine-related polymorphisms and fear memory reconsolidation using a three-day FC paradigm (for a review of this paradigm, see [138]). The authors focused on a reactivation procedure (CS+ presentations without US on day 2), which triggers the reconsolidation of the previously learned fear memories (acquired on day 1 of the experiment). Participants underwent fear extinction either within a 10-min window after reactivation or outside the reconsolidation time window (after 6 h). Interestingly, S allele carriers who performed extinction outside the reconsolidation period showed an increased response to CS + during re-acquisition on day 3 [137]. This study provides evidence for the role of 5-HT gene polymorphism in fear memory re-acquisition and reconsolidation, emphasizing the importance of the timing of extinction with respect to the reconsolidation window, especially for S allele carriers. 

Hartley et al. [31] investigated genetic variations related to the 5-HTT polyadenylation polymorphism (STPP). Carriers of the G allele polymorphism exhibited reduced functioning of the 5-HTT, leading to increased availability of 5-HT in the synaptic cleft. The study utilized a two-day FC paradigm. On the first day, fear acquisition and extinction phases were performed, and on the second day, another extinction session was conducted to assess the retention of extinction learning or spontaneous recovery of the previous aversive learning. The authors reported a linear increase in spontaneous fear recovery, trait anxiety, and depressive symptoms as a function of the overall number of the risk alleles G of STPP. Interestingly, a similar pattern was observed in knockout mice, further supporting the significant impact of this polymorphism on fear extinction [31]. In a classical conditioning paradigm, Hermann et al. [88] demonstrated that carriers of the S allele exhibited greater activation of the insula, a relevant brain region in FC. This increased activation was observed both in response to CS during late acquisition and in response to US. Additionally, carriers of the S allele who experienced a higher number of traumatic events showed a lower response of the amygdala during extinction. These individuals also displayed greater expression of fear responses and reduced activation in the left amygdala during extinction. Furthermore, carriers of the T allele of the TPH2 polymorphism, which affects 5-HT synthesis, exhibited enhanced responsiveness in the amygdala during acquisition and in the vmPFC during extinction. Notably, the combined influence of the two polymorphisms was associated with higher responses in dACC during extinction. Although the 5-HTTLPR genotype did not have a significant effect on differentially conditioned SCR, S allele carriers with a higher number of traumatic life events exhibited enhanced differential SCR during late acquisition. This emphasizes the importance of gene and environment interactions for a detectable outcome in peripheral physiological responses [139].

Klucken et al. [140] also investigated the impact of genetic and environmental factors on FC. In their study, participants were genotyped for 5-HTT-related genetic variations and subjected to a FC paradigm with SCR assessment. The study also utilized fMRI to evaluate individual differences in cerebral activity during FC. The results showed that S allele carriers exhibited a greater differential response to CS+ compared to CS- in brain areas involved in fear learning, including the right amygdala, left thalamus, bilateral insula, and bilateral occipital cortex. However, no significant differences were found in the SCR. Additionally, carriers of the S allele with a higher number of stressful life events showed hyperactivity in response to CS+ in the insula and occipital cortex [140].

Glotzbach-Schoon et al. [141] investigated the influence of 5-HTT polymorphism and the neuropeptide S (NPS), which has been reported to affect arousal, fear, and anxiety responses, on potential vulnerability to anxiety disorders. The research involved the context of the FC paradigm in a virtual reality environment. The results revealed that individuals carrying both the S and T alleles of the 5-HTTLPR and NPSR1 polymorphisms exhibited a stronger FC response to anxiety-inducing context during the late acquisition phase. This suggests the role of 5-HT polymorphisms as possible predictors of anxiety disorder development.

Furthermore, the authors observed that participants with both S+/T+ (5-HTT/NPS) alleles demonstrated extinction of the conditioned response 24 h after consolidation, indicating improved fear learning and extinction. Conversely, participants with only one S allele (5-HHT) showed continued discrimination for the CS during extinction [141]. Moreover, the NPSR1 polymorphism seemed to modulate implicit but not explicit learning of fear, suggesting that the 5-HT system may influence amygdala-dependent learned fear but not explicit assessment of threatening contexts. 

Heitland et al. [142] investigated the potential influence of 5-HTT and corticotropin-releasing factor (CRF) polymorphisms on fear acquisition and expression. The participants underwent a FC paradigm in a virtual reality environment, and the conditioned response was measured using the eyeblink startle reflex. The results revealed that participants carrying both the G allele of CRF and the S allele of 5-HTT showed an increase in uninstructed cue-driven fear acquisition. This effect was observed only for uninstructed fear acquisition and not after explicit instructions on the contingency of the conditioning paradigm (i.e., instructed FC). This suggests that the combination of these two genetic variations influences FC at the level of defensive reflexes rather than at the cognitive level [142]. Notably, considering the CRF polymorphism, participants with C/G heterozygotes did not exhibit conditioning to the threat cue, whereas such conditioning was present in C/C homozygotes. This finding highlights the importance of both 5-HTT and CRF polymorphisms in fear learning and expression. 

In a revision of the same investigation [143], scholars reported an additional interaction between CRHR1 and the triallelic 5-HTTLPR variant in relation to cued fear acquisition, contrasting with earlier results. The revisited study confirmed previous findings concerning the CRHR1 G allele but revealed an additional interaction with another variant of the 5-HTT polymorphism.

In a clinical multicenter trial by Straube et al. [144], researchers explored the impact of the HTR1A 5-HT receptor on FC using fMRI in a clinical sample of panic disorder patients with agoraphobia. The study found that GG carriers exhibited increased activity in the amygdala and hippocampus in response to threat and safety cues both before and after cognitive behavioral therapy [144].

Subsequently, Klumpers et al. [145] investigated how 5-HTT-related genetic variations can influence brain and physiological activity during threat anticipation, using a classical conditioning paradigm. The results demonstrated a mediating effect of dorsomedial prefrontal cortex (dmPFC) in threat signal processing, with greater activation in S allele carriers in both uninstructed (classic) and instructed FC paradigms. The study highlights how a genetic variation in the 5-HTT gene affects dmPFC activity during fear acquisition, observable through SCR or startle response [145]. 

The study conducted by Baas and Heitland [146] investigated the relationship between 5-HTR1A gene polymorphism and individual variability in contextual fear response in humans using a contextual FC paradigm. The results showed that individuals carrying the C allele of the 5-HTR1A gene displayed an increased contextual fear response, measured by fear-potentiated startle. Additionally, concerning subjective fear ratings, the polymorphism significantly affected fearful responses to the cue but not to the context, indicating a dissociation between physiological measures and subjective ratings [146].

In another clinical study by Wannemueller et al. [147], researchers hypothesized that S allele carriers suffering from phobia might exhibit different response to exposure-based treatments. The study involved verbally assessing the level of fear related to phobic stimuli at different time points (before, immediately after treatment and at 7 months follow up). Participants then underwent standardized exposure treatments for different types of phobia. The results showed that the SS carriers exhibited reduced fears immediately after treatment, similarly to LL carriers. However, in the follow-up session, SS allele carriers exhibited a strong return of fear compared to LL carriers [147], suggesting that in the long term, they may struggle to inhibit responses to previously threatening stimuli.

Finally, Schipper et al. [148] conducted a study to investigate fear learning, specifically bradycardia (a measure of heart rate) associated with freezing behavior, in both animals and humans, while also considering the role of the 5-HTT polymorphism. The study revealed an association between 5-HTT availability and parasympathetically-mediated bradycardia during threat anticipation for CS. In human individuals with the 5-HTTLPR S allele carriers and 5-HTT knockout rats, the response to the CS+ threat was particularly pronounced and consisted of a heightened bradycardic reaction. Additionally, researchers observed reduced mPFC activation and increased threat-related connectivity between the amygdala and periaqueductal gray (PAG) in these individuals. These findings suggest that the 5-HTTLPR polymorphism may influence the way the brain processes threat cues, with S allele carriers displaying a stronger fear response than LL carriers [148], potentially indicating an increased risk for anxiety disorders.

Collectively, these data have highlighted the effect of 5-HT-related gene polymorphisms on aversive learning. Carriers of the 5-HTT S allele exhibit enhanced social fear learning, while C carriers show increased contextual fear responses, providing supporting evidence of the role of 5-HT in fear learning. Moreover, these results suggest a broader social influence of 5-HT-related polymorphisms (especially the S and T alleles) on human behavior, potentially contributing to the development of maladaptive aversive associations and, in turn, increasing the risk of anxiety disorders. Refer to Table 3 for a summary of studies on gene polymorphism.

## 4. Discussion

In this review, a detailed examination of the findings reveals the significance of 5-HT levels in the brain for various aspects of FC. Researchers have explored two main approaches: first, they experimentally manipulated 5-HT levels using SSRIs or other 5-HT receptor agonist/antagonist agents, or by depleting the 5-HT precursor Tryptophan. Second, they investigated the effects of natural variations in 5-HT/T or 5-HT-related gene polymorphisms on FC and fear memory. The overall results suggest a link between higher 5-HT levels and enhanced fear learning in humans.

Numerous studies have emphasized the importance of specific brain regions in FC, particularly the amygdala [91,92], hippocampus [149], PFC [34,50,76,88,150,151,152,153], insula [81,139], and anterior cingulate cortex [89]. These regions house significant populations of 5-HT receptors [101,102,103,104,154,155], providing a strong rationale to explore the impact of 5-HT on fear learning. Additionally, studies have revealed a genetic influence on 5-HT levels in the brain, with carriers of a specific polymorphism (S alleles) exhibiting higher 5-HT levels [156,157]. Moreover, evidence suggests that contextual FC is modulated by an interaction between 5-HTTLPR polymorphisms and the NPS receptor gene [141]. These findings complete the puzzle, proving a solid basis for investigating the relationship between genes and fear-related behavior, mediated through 5-HT levels in the brain. 

### 4.1. SSRI Studies

SSRIs are the first-choice treatment for anxiety and mood disorders [122,158,159]. They work by selectively or exclusively blocking the reuptake of 5-HT [160], thus increasing its availability in the synaptic cleft and elevating overall 5-HT concentration. The level of 5-HT in the brain leads to various behavioral and physiological effects in both animals [3] and humans [161], particularly in the context of FC [125]. On this basis, SSRIs have been used as experimental manipulations in some of the reviewed articles using different types of FC paradigms (for a comprehensive review of different FC paradigms, see [57]). In this context, it is important to take into account the mode of administration for 5-HT modulators, specifically distinguishing between acute and chronic administration. 

Studies have shown that acute administration of citalopram impacts fear potentiated startle to threat cues and sustained potentiated startle [122], which serves as an experimental model of anxiety. However, subchronic administration (i.e., for two weeks) of citalopram [123] did not affect fear potentiated startle, aligning with the other studies that reported no effect of subchronic administration of another SSRI, escitalopram, on fear acquisition [124]. Instead, subchronic citalopram decreased sustained startle potentiation, which is in line with the therapeutic effects of SSRIs on anxiety symptoms but not fear symptoms. This dissociation can be attributed, at least in part, to different neural bases in the brain regulating these two phenomena: cued fear is regulated by the CeA, whereas sustained contextual anxiety is regulated by the BNST. 

However, in pioneering studies within this research area, acute administration of FEN (a 5-HT promoter) showed no effects on the conditioned response and resulted in decreased anxiety, as evidenced by fewer SCR fluctuations [119]. Another study reported that ritanserin administration, a 5-HT2 antagonist, either interfered with the learning process by suppressing the expression of learned fear or facilitated the extinction process [118]. Nevertheless, it is important to consider that the paradigm used in that study involved single-trial conditioning, requiring cautious interpretation of the results concerning the role of 5-HT in learning. However, another study utilizing the same paradigm but with different drugs (buspirone, fluvuxamine, and diazepam) yielded comparable results [120]. Furthermore, a relatively recent investigation examining the effects of different SSRIs [128] found no impact of SSRIs on anxiety, as measured by the startle response, in a clinical sample. 

In studies using 5-HT modulating drugs, there is significant variation in paradigms, types of drugs, and measures of learning or extinction. are sometimes widely different and do not allow for direct comparisons of the results. For example, acute administration of citalopram can enhance cortisol levels [162], and since cortisol may facilitate fear learning in the amygdala and the BNST [163], it becomes important to discuss the effects of a 5-HT promoting drug in the context of their interactions with other neurotransmitters or hormones. This complexity is further compounded when considering studies that use different types of SSRIs [128], as different drug manipulations can potentially affect hormonal and neurotransmitter dynamics in the brain differently. This highlights the necessity for further detailed investigations into the mechanisms of action of each specific 5-HT agonist/antagonist, particularly in the context of anxiety and fear. Emphasizing the importance of a reliable and comprehensive experimental procedure is crucial to capturing the intricate phenomenon of learning mediated by 5-HT.

ATD is a widely used experimental manipulation to investigate the role of 5-HT. In this method, tryptophan, the precursor molecule of 5-HT, is temporarily removed from the diet to reduce 5-HT synthesis in the brain [130,164,165,166]. A pioneering study employing ATD to examine fear learning reported no significant effects on phasic cued conditioning. However, ATD has been shown to disrupt fear acquisition on a neural and physiological level, as evidenced by fMRI and SCR [127], and it has been associated with increased anxiety as measured by the startle response [130]. These results are consistent with findings by Grillon et al. [122] concerning anxiety, revealing a reverse pattern when 5-HT levels are extinguished rather than promoted. Additionally, the study highlights the importance of the measures adopted (startle response versus imaging and SCR) in capturing the phenomenon of learning. 

Furthermore, a recent study demonstrated that administering ATD one day after conditioning significantly reduced the fear response during spontaneous recovery and also attenuated the expression of previously acquired fear during this phase [132]. This finding aligns with the results of Grillon et al. [122], where a reduction in 5-HT through ATD led to a decrease in fear expression during spontaneous recovery, whereas an increase in 5-HT facilitated fear expression. Notably, Grillon et al. [122] employed instructed fear conditioning and concluded that acute citalopram administration facilitated fear expression rather than fear learning or recognition. Indeed, the participants were fully informed about the contingencies beforehand and could even see them on a monitor during the experiment. However, it is important to acknowledge that making a direct comparison between these findings might be challenging due to different methodologies and experimental designs.

### 4.2. Genetic Studies

Studies in behavioral genetics suggest that approximately 30% of the variations in human FC [167] and the development of anxiety disorders [168] can be attributed to genetic factors. While environmental factors, such as life-threatening experiences, have been shown to increase the risk of anxiety disorders [169,170], genetic predispositions can also influence fear learning, affecting acquisition and suppression processes [171]. In this context, several studies have explored the impact of 5-HT-associated genes on fear-related processes like acquisition, extinction, and memory (encoding, retrieval, etc.) in humans [54,167,172]. 

A prominent candidate investigated in numerous studies, akin to evidence from animal models (for a review, see [54]), is the polymorphism in the promoter region of the 5-HT transporter gene (5-HTTLPR). The 5-HTT plays a crucial role in regulating 5-HT in the brain by removing 5-HT from the synaptic cleft [156]. In humans, the expression of the short (S) allele of 5-HTT is associated with a partial transcription of the long (L) allele, resulting in lower functionality of 5-HTT and consequently higher 5-HT concentration in the extracellular area [157]. Moreover, the S allele appears to be associated with increased amygdala activity in response to emotional stimuli and higher trait anxiety [172,173]. Correspondingly, carriers of the 5-HTT S allele tend to report heightened trait and state anxiety [157,174,175,176,177]. 

Considering this rationale, most reviewed studies have shown a significant influence of 5-HT gene polymorphisms on fear learning and memory. In general, individuals with a higher availability of 5-HT, due to the expression of the S allele, exhibit greater emotional reactivity to threatening stimuli. However, it is important to consider some differences among the reported studies. The variability arises from differences in methodologies, including outcome measures and experimental design (e.g., sample selection), contributing to the relative inconsistency of some results (for a review of FC methodologies, refer to [57]). 

Some of the studies reviewed found that 5-HTTLPR genotype influenced FC, as measured either by the startle response, brain imaging, or both methods, but did not influence SCR [134,139,140]. Conversely, other studies reported an effect of 5-HTT genetic variations on FC using SCR [133,135]. The disparity in these results could be attributed to the fact that genetic variations of 5-HTT may influence more fundamental aspects of fear processing, which are detected by the startle response but not by SCR. The startle response likely represents an ancestral automatic reaction to threats shared across various species, reflecting activation of an innate defensive system directly connected to the amygdala. Therefore, it serves as a relevant measure for automatic and implicit fear learning. In contrast, SCR appears to be more sensitive to higher-level cognitive understanding of the contingencies involved in the aversive learning paradigm [134,139]. 

Interestingly, some studies have not only demonstrated the effects of 5-HT-related gene polymorphisms [31,133,142] but also other types of genetic variations, possibly even their interaction [134], on aversive learning. The findings of Crişan et al. [135], showing that carriers of the 5-HTT S allele exhibit improved social fear learning, extend prior evidence on the role of 5-HT in individual learning to observational learning. This implies a broader social impact of 5-HT-related polymorphisms on human behavior, which could contribute to the development of maladaptive aversive associations, potentially leading to anxiety disorders [178,179,180,181].

As a final point in this section, it is essential to underscore once again the significant and dynamic interaction between genes and the environment. On the one hand, evidence points toward genes exerting an influential role in brain function and behavior related to fear learning [31,133,134,135,136,137,141]. On the other hand, environmental factors, such as traumatic experiences, have demonstrated the ability to influence gene expression, either enhancing or suppressing it [139,140,182]. In summary, both genetic factors and the environment play crucial roles in shaping the processes of fear learning and its behavioral outcomes, and their interplay should be taken into account to fully comprehend the complexities of this phenomenon.

### 4.3. Sex, Serotonin, and FC

The studies analyzed in this review highlight the significant role of 5-HT in modulating threat learning. However, it is important to acknowledge that other factors may also influence the effects of 5-HT on fear learning and expression. Notably, a substantial body of literature, mainly focusing on animal studies, has shown an interaction between 5-HT, aversive learning, and sex differences [183,184,185,186,187]. While providing an extensive description of these animal studies is beyond the scope of this review, it is relevant to outline certain aspects regarding the potential involvement of sex in the context of the discussed human studies. 

Overall, the findings presented in this article, irrespective of the specific 5-HT manipulations employed, demonstrate inconsistent and mixed results concerning the potential impact of sex on the reported effects. Most of the reviewed studies did not distinguish between the responses of female and male participants [119,124,125,131,133,137,144,148]. Additionally, some studies used male-only samples [127,140] or samples that were not balanced for sex [121]. Furthermore, certain studies have shown no significant effect of sex on the interaction between 5-HT and fear learning processes [123,132,135]. Lastly, the limited number of studies reporting gender differences in the impact of 5-HT modulators on fear learning pose challenges for comparison, primarily stemming from the utilization of outdated experimental paradigms and the inclusion of generally small or heterogeneous samples [118,120,121]. 

Given the potential role of sex in fear learning, as indicated by the aforementioned animal studies, it is recommended that future human studies systematically and comparatively examine the relations between sex, 5-HT, and emotional learning. Understanding how sex may influence these processes holds significant clinical importance, especially considering the asymmetrical impact of certain psychopathologies on females and males [183,188]. Such insights could pave the way for the development of targeted therapies tailored to individual needs and gender-specific responses, ultimately enhancing treatment effectiveness and patient outcomes.

## 5. Conclusions and Future Directions

To explore the complex relationship between normal and abnormal behavior and its link to brain functioning, a comprehensive and multidimensional perspective is necessary. The studies reviewed here have employed diverse experimental approaches to investigate the interplay of specific genetic variations, interactions with environmental factors, brain activations, and behavior in the context of fear learning and memory in humans. For instance, they reveal how carriers of a particular gene combination may become more susceptible to anxiety or fear-related behaviors when exposed to identical traumas or environmental influences. Although highly informative for clinical populations and understanding the mechanisms underlying the development of fear and anxiety-related psychopathologies, research on gene–environment interactions remains relatively scarce, warranting further exploration.

Additionally, it is essential to further investigate the mechanisms by which different SSRIs produce their effects. Investigations of this nature have the potential to provide valuable insights that could have significant implications for clinical research. In this context, it may be beneficial to distinguish the role of 5-HT in encoding and consolidation memory processes, two distinct stages of the learning process [189], as this distinction holds significance for tailoring therapeutic approaches and optimizing interventions for fear-related disorders.

Other directions for future research involve the integration of further methodological approaches into FC protocols. For example, it would be relevant to incorporate measures of disgust processing/disgust sensitivity, given the evidence linking 5-HT to this affective/interoceptive experience [190,191,192,193], and this could enhance our understanding of processes interlinked with affective learning, such as moral decision-making [194,195,196], and predict emotional reactions to negative outcomes [197,198,199,200,201,202].

The advancement of virtual reality as a tool to study emotions and behavioral reactions holds promise for investigating FC in controlled and ecologically valid contexts [38,141,203,204]. Integrating experiments conducted in virtual environments with genotyping techniques or other experimental manipulations of the serotonergic system could provide valuable insights into the role of 5-HT in various processes. 

Finally, given the potential to impact fear memories using techniques such as transcranial magnetic stimulation and transcranial electrical stimulation [34,88], the trajectory of future FC research could effectively integrate state-of-the-art brain stimulation protocols. These techniques could be used to map and regulate cortico-cortical interactions between key cortical regions [205,206,207,208,209]. This would enrich the pharmacological and genetic approaches discussed in this systematic review, ultimately fostering the establishment of robust causal links between FC neural networks and 5-HT activity in fear learning processes. These techniques hold significant promise not only for advancing our understanding of these links but also for potential clinical applications [210,211,212,213,214], particularly in the realm of anxiety disorders [27,152,153,215,216,217].

In conclusion, integrating these methodologies comprehensively within FC research could help us further investigate the relevance of the neurotransmitter 5-HT in fear learning and memory across both experimental and clinical settings. This integrative approach has the potential to enhance our understanding of the mechanisms behind affective learning and also provide deeper insights into a range of psychopathologies, such as PTSD and anxiety disorders. Ultimately, this research could inform the development of more effective treatment strategies that are tailored to the distinctive characteristics of these mental health conditions.

## Figures and Tables

**Table 1:** Summary of studies on SSRIs or other 5-HT-modulating drugs and fear learning. ** = Studies without SSRI administration but involving 5-HT level evaluation by PET scan.

Study	Design	Outcome Measure(s)	Type of Manipulation	Effect of Manipulation	N (Male/Female)	Type of CS/US	Brief Statement of Findings
Hensman et al. (1991) [118]	Between group double blind	SCR	Ritanserin (10 mg)	5-HT antagonist	30 (15/15)	Tone/White noise	Reduced SCR amplitude and spontaneous fluctuations in the extinction phase
Hetem et al. (1996) [119]	Between group double blind	SCR	d-fenfluramine (15 mg or 30 mg)	5-HT agonist	42 (27/15)	Tone/White noise	Decreased anxiety during a public speaking task but no effects on FC
Hellewell et al. (1999) [120]	Between group single blind	SCR	(a) Buspirone (5 mg), (b) Diazepam (2 mg), (c) Fluvoxamine (25 mg)	(a) Azapirone (b) Benzodia-zepine (c) SSRI	50 (25/25)	Tone/White noise	Decreased SCR in both habituation and extinction
Silva et al. (2001) [121]	Between group double blind	SCR	Nefazodone (100 mg or 200 mg)	SSRI	29 (9/20)	Tone/White noise	Decreased level of SCR fluctuation in both habituation and extinction
Grillon et al. (2007) [122]	Within group Double blind cross over	Eye blink startle response	Single dose of Citalopram (20 mg)	SSRI	18 (9/9)	Geometric colored shapes/Electric Shock	Increased phasic and sustained fear-potentiated startle
Grillon et al. (2008) [123]	Between group double blind	Eye blink startle response	Two weeks of Citalopram (20 mg)	SSRI	28 (19/9)	Geometric colored shapes/Electric shock	Decreased sustained anxiety but not phasic cued fear potentiation
Bui et al. (2012) [124]	Double blind randomized placebo controlled	SCR	Two weeks of Escitalopram (10 mg)	SSRI	38 (23/15)	Yellow circle, white square/Electric shock	Facilitation of extinction learning but not acquisition
Åhs et al. (2015) [125]	Within group	SCR PET	NA **	NA	16 (8/8)	Geometric shapes/Electric shock	Lower 5-HTT in amygdala, insula, and dorsal anterior cingulate cortex predicts increased SCR during fear learning
Gorka et al. (2017) [128]	Mixed group	Eye blink startle response	Two weeks of Sertraline, Fluoxetine, Paroxetine, Escitalopram, or Citalopram (variable dose)	SSRI	72 (52/20)	Geometric colored shapes/Electric shock	No effect of SSRI administration on magnitude of startle response

**Table 2:** Tryptophan depletion studies.

Study	Design	Outcome Measure(s)	Type of Manipulation	N (Male/Female)	Type CS/US	Brief Statement of Findings
Robinson et al. (2012) [131]	Within group	Eye blink startle response	Dietary	20 (13/7)	Geometric shapes/Electric shock	Increased startle potentiation to contextual threat (sustained anxiety) but not explicit threat cues (cued fear)
Hindi Attar et al. (2012) [127]	Mixed design Between subject Double blind	SCR fMRI	Dietary	39 (39/0)	Geometrical shapes/Increased temperature	Decreased fear response during fear acquisition in both fMRI and autonomic responses
Kanen et al. (2021) [132]	Between group double blind	SCR	Dietary	47 (29/18)	Geometrical shapes/Electric shock	Decreased fear response in spontaneous recovery

**Table 3:** Summary of studies on gene polymorphisms. *** = Virtual reality.

Study	Outcome Measure(s)	Polymor-phism	N (Male/Female)	Type of CS/US	Brief Statement of Findings
Garpenstrand et al. (2001) [133]	SCR	5-HTT MAOA	40 (14/26)	Geometric shapes/Electric shock	Both 5-HTT and MAOA polymorphisms could explain variation in fear learning (S allele carriers showed increased fear response during acquisition)
Lonsdorf et al. (2009) [134]	Eye blink startle response SCR	5-HTT	48 (25/23)	Face stimuli/Electric shock	Only carriers of S allele exhibited conditioned startle potentiation in both acquisition and extinction
Crisan et al. (2009) [135]	SCR Electrocardiogram	5-HTT	32 (9/23)	Geometric shape/Electric shock	Increased fear response during social FC task in 5-HT S-carriers
Klumpers et al. (2011) [136]	Eye blink startle response	5-HTT	82 (22/62)	Face stimuli/Electric shock	S carriers showed stronger FPS during fear learning but no deficit in fear downregulation
Agren et al. (2012) [137]	SCR	5-HTT COMT	66 (28/38)	Images of colorful lamps/Electric shock	S allele carriers who underwent extinction outside reconsolidation time window showed an increased fear response in the reacquisition phase (day 3).
Hartley et al. (2012) [31]	SCR	STPP	141 (54/87)	Geometric shapes/Electric shock	Increased spontaneous fear recovery and impaired extinction retention in G allele carriers of STPP
Hermann et al. (2012) [88]	SCR fMRI	5-HTT TPH2	74 (37/37)	Geometric shapes/Electric shock	Increased Insula activation in response to both CS and US in S allele carriers
Klucken et al. (2013) [140]	SCR fMRI	5-HTT	47 (47/0)	Geometric shapes/Aversive pictures	Greater response to CS+ in right amygdala, left thalamus, bilateral insula and bilateral occipital cortex in S allele carriers
Glotzbach-Schoon et al. (2013) [141]	Eye blink startle response SCR	5-HTT NPSR1	80 (31/49)	Context ***/Electric shocks	Only carriers of both S+/T+ alleles showed higher startle responses in acquisition
Heitland et al. (2013) [142]	Eye blink startle response	5-HTT CRHR1	146 (59/87)	Context ***/Electric shock	Increased FPS to threat context in participants carrying both G allele and S allele during uninstructed FC
Heitland et al. (2016) [143]	Eye blink startle response	5-HTT CRHR1	205 (59/87)	Context ***/Electric shock	Additional interaction between CRHR1 and triallelic 5-HTTLPR variant on acquisition
Straube et al. (2014) [144]	fMRI SCR	HTR1A	39 (13/26)	Geometric shapes/White noise	Increased activation of the amygdala and hippocampus in response to fear cues and safety cues before and after treatment in GG carriers
Klumpers et al. (2015) [145]	SCR Eye blink startle response fMRI	5-HTT	168 (120–48)	Geometric shapes, face stimuli/Electric shock	Fear related dmPFC was increased in S allele carriers during threat learning
Baas & Heitland (2015) [146]	Eye blink startle response	5-HTT	150 (60/90)	Context ***/Electric shock	Enhanced contextual FPS response in C-carriers
Wannemueller et al. (2018) [147]	Self-reports	5-HTT	157 (25/132)	Fear mental images	Phobic S allele carriers showed greater return of fear 7 months after undergoing exposure-based phobia treatments
Schipper et al. (2019) [148]	Heart rate	5-HTT	104 (104/0)	Visual stimuli/Electric shock	S allele carriers displayed significantly stronger fear bradycardia to CS+

## Data Availability

Not applicable.

## References

[1] Erland L.A.E., Turi C.E., Saxena P.K., Pilowsky P.M. (2019). Serotonin in plants: Origin, functions, and implications. Serotonin the Mediator That Spans Evolution.

[2] Azmitia E.C. (2001). Modern views on an ancient chemical: Serotonin effects on cell proliferation, maturation, and apoptosis. Brain Res. Bull..

[3] Bacqué-cazenave J., Bharatiya R., Barrière G., Delbecque J.-P.P., Bouguiyoud N., Di Giovanni G., Cattaert D., De Deurwaerdère P. (2020). Serotonin in animal cognition and behavior. Int. J. Mol. Sci..

[4] Moses L., Mohammad-Zadeh L.F., Moses L., Gwaltney-Brant S.M. (2008). Serotonin: A review. J. Vet. Pharmacol. Ther..

[5] Deakin J.F.W. (2013). The origins of ‘5-HT and mechanisms of defence’ by Deakin and Graeff: A personal perspective. J. Psychopharmacol..

[6] Miyazaki K.W., Miyazaki K., Doya K. (2012). Activation of dorsal raphe serotonin neurons is necessary for waiting for delayed rewards. J. Neurosci..

[7] Branchi I. (2011). The double edged sword of neural plasticity: Increasing serotonin levels leads to both greater vulnerability to depression and improved capacity to recover. Psychoneuroendocrinology.

[8] Puglisi-Allegra S., Andolina D. (2015). Serotonin and stress coping. Behav. Brain Res..

[9] Edwards D.H., Spitzer N. (2006). Social Dominance and Serotonin Receptor Genes in Crayfish. Curr. Top. Dev. Biol..

[10] Stevenson P.A., Rillich J. (2012). The decision to fight or flee—Insights into underlying mechanism in crickets. Front. Neurosci..

[11] Miczek K.A., Fish E.W., De Bold J.F., De Almeida R.M. (2002). Social and neural determinants of aggressive behavior: Pharmacotherapeutic targets at serotonin, dopamine and γ-aminobutyric acid systems. Psychopharmacology.

[12] Yeh S.R., Fricke R.A., Edwards D.H. (1996). The effect of social experience on serotonergic modulation of the escape circuit of crayfish. Science.

[13] Huber R., Smith K., Delago A., Isaksson K., Kravitz E.A. (1997). Serotonin and aggressive motivation in crustaceans: Altering the decision to retreat. Proc. Natl. Acad. Sci. USA.

[14] Bacqué-Cazenave J., Cattaert D., Delbecque J.P., Fossat P. (2018). Alteration of size perception: Serotonin has opposite effects on the aggressiveness of crayfish confronting either a smaller or a larger rival. J. Exp. Biol..

[15] LeDoux J. (2003). The emotional brain, fear, and the amygdala. Cell. Mol. Neurobiol..

[16] LeDoux J.E. (2014). Coming to terms with fear. Proc. Natl. Acad. Sci. USA.

[17] Battaglia S., Nazzi C., Thayer J.F. (2023). Fear-induced bradycardia in mental disorders: Foundations, current advances, future perspectives. Neurosci. Biobehav. Rev..

[18] Battaglia S. (2022). Neurobiological advances of learned fear in humans. Adv. Clin. Exp. Med..

[19] Milad M.R., Quirk G.J. (2012). Fear extinction as a model for translational neuroscience: Ten years of progress. Annu. Rev. Psychol..

[20] Vervliet B., Baeyens F., Van den Bergh O., Hermans D. (2013). Extinction, generalization, and return of fear: A critical review of renewal research in humans. Biol. Psychol..

[21] Davis L.L., Suris A., Lambert M.T., Heimberg C., Petty F. (1997). Post-traumatic stress disorder and serotonin: New directions for research and treatment. J. Psychiatry Neurosci..

[22] Indovina I., Robbins T.W., Núñez-Elizalde A.O., Dunn B.D., Bishop S.J. (2011). Fear-Conditioning Mechanisms Associated with Trait Vulnerability to Anxiety in Humans. Neuron.

[23] Parsons R.G., Ressler K.J. (2013). Implications of memory modulation for post-traumatic stress and fear disorders. Nat. Neurosci..

[24] Tortella-Feliu M., Fullana M.A., Pérez-Vigil A., Torres X., Chamorro J., Littarelli S.A., Solanes A., Ramella-Cravaro V., Vilar A., González-Parra J.A. (2019). Risk factors for posttraumatic stress disorder: An umbrella review of systematic reviews and meta-analyses. Neurosci. Biobehav. Rev..

[25] Vicario C.M., Felmingham K.L. (2018). Slower time estimation in post-traumatic stress disorder. Sci. Rep..

[26] Vicario C.M., Martino G., Lucifora C., Felmingham K. (2022). Preliminary evidence on the neural correlates of timing deficit in post-traumatic stress disorder. Eur. J. Psychotraumatol..

[27] Jafari E., Alizadehgoradel J., Koluri F.P., Nikoozadehkordmirza E., Refahi M., Taherifard M., Nejati V., Hallajian A.-H., Ghanavati E., Vicario C.M. (2021). Intensified electrical stimulation targeting lateral and medial prefrontal cortices for the treatment of social anxiety disorder: A randomized, double-blind, parallel-group, dose-comparison study. Brain Stimul..

[28] Vicario C.M., Scavone V., Lucifora C., Falzone A., Pioggia G., Gangemi S., Craparo G., Martino G. (2023). Evidence of abnormal scalar timing property in alexithymia. PLoS ONE.

[29] La Rosa V.L., Gori A., Faraci P., Vicario C.M., Craparo G. (2022). Traumatic distress, alexithymia, dissociation, and risk of addiction during the first wave of COVID-19 in Italy: Results from a cross-sectional online survey on a non-clinical adult sample. Int. J. Ment. Health Addict..

[30] Asberg M., Thoren P., Traskman L., Bertilsson L., Ringberger V. (1976). “Serotonin depression”—A biochemical subgroup within the affective disorders?. Science.

[31] Hartley C.A., McKenna M.C., Salman R., Holmes A., Casey B.J., Phelps E.A., Glatt C.E. (2012). Serotonin transporter polyadenylation polymorphism modulates the retention of fear extinction memory. Proc. Natl. Acad. Sci. USA.

[32] Kahn R.S., Van Praag H.M., Wetzler S., Asnis G.M., Barr G. (1988). Serotonin and anxiety revisited. Biol. Psychiatry.

[33] Castrén E. (2005). Is mood chemistry. Nat. Rev. Neurosci..

[34] Borgomaneri S., Battaglia S., Garofalo S., Tortora F., Avenanti A., di Pellegrino G. (2020). State-dependent TMS over prefrontal cortex disrupts fear-memory reconsolidation and prevents the return of fear. Curr. Biol..

[35] Borgomaneri S., Battaglia S., Avenanti A., di Pellegrino G. (2021). Don’t Hurt Me No More: State-dependent Transcranial Magnetic Stimulation for the treatment of specific phobia. J. Affect. Disord..

[36] Borgomaneri S., Battaglia S., Sciamanna G., Tortora F., Laricchiuta D. (2021). Memories are not written in stone: Re-writing fear memories by means of non-invasive brain stimulation and optogenetic manipulations. Neurosci. Biobehav. Rev..

[37] Pittig A., Treanor M., LeBeau R.T., Craske M.G. (2018). The role of associative fear and avoidance learning in anxiety disorders: Gaps and directions for future research. Neurosci. Biobehav. Rev..

[38] Lucifora C., Grasso G.M., Nitsche M.A., D’Italia G., Sortino M., Salehinejad M.A., Falzone A., Avenanti A., Vicario C.M. (2022). Enhanced fear acquisition in individuals with evening chronotype. A virtual reality fear conditioning/extinction study. J. Affect. Disord..

[39] Hartley C.A., Phelps E.A. (2010). Changing fear: The neurocircuitry of emotion regulation. Neuropsychopharmacology.

[40] Bunney W.E., Davis J.M. (1965). Norepinephrine in depressive reactions: A review. Arch. Gen. Psychiatry.

[41] Coppen A. (1967). The biochemistry of affective disorders. Br. J. Psychiatry.

[42] Wong M.-L., Licinio J. (2004). From monoamines to genomic targets: A paradigm shift for drug discovery in depression. Nat. Rev. Drug Discov..

[43] Bertini C., Làdavas E. (2021). Fear-related signals are prioritised in visual, somatosensory and spatial systems. Neuropsychologia.

[44] Borgomaneri S., Gazzola V., Avenanti A. (2015). Transcranial magnetic stimulation reveals two functionally distinct stages of motor cortex involvement during perception of emotional body language. Brain Struct. Funct..

[45] Borgomaneri S., Vitale F., Battaglia S., Avenanti A. (2021). Early right motor cortex response to happy and fearful facial expressions: A TMS motor-evoked potential study. Brain Sci..

[46] Borhani K., Ladavas E., Maier M.E., Avenanti A., Bertini C. (2015). Emotional and movement-related body postures modulate visual processing. Soc. Cogn. Affect. Neurosci..

[47] Tamietto M., De Gelder B. (2010). Neural bases of the non-conscious perception of emotional signals. Nat. Rev. Neurosci..

[48] Lonsdorf T.B., Merz C.J. (2017). More than just noise: Inter-individual differences in fear acquisition, extinction and return of fear in humans—Biological, experiential, temperamental factors, and methodological pitfalls. Neurosci. Biobehav. Rev..

[49] Lonsdorf T.B., Merz C.J., Fullana M.A. (2019). Fear extinction retention: Is it what we think it is?. Biol. Psychiatry.

[50] Battaglia S., Harrison B.J., Fullana M.A. (2022). Does the human ventromedial prefrontal cortex support fear learning, fear extinction or both? A commentary on subregional contributions. Mol. Psychiatry.

[51] Vicario C.M. (2013). Uncovering the neurochemistry of reward and aversiveness. Front. Mol. Neurosci..

[52] Vicario C.M. (2014). Aberrant disgust response and immune reactivity in cocaine-dependent men might uncover deranged serotoninergic activity. Front. Mol. Neurosci..

[53] Vicario C.M., Rafal R.D., Martino D., Avenanti A. (2017). Core, social and moral disgust are bounded: A review on behavioral and neural bases of repugnance in clinical disorders. Neurosci. Biobehav. Rev..

[54] Bauer E.P. (2015). Serotonin in fear conditioning processes. Behav. Brain Res..

[55] Cools R., Roberts A.C., Robbins T.W. (2008). Serotoninergic regulation of emotional and behavioural control processes. Trends Cogn. Sci..

[56] LeDoux J.E. (2000). Emotion circuits in the brain. Annu. Rev. Neurosci..

[57] Lonsdorf T.B., Menz M.M., Andreatta M., Fullana M.A., Golkar A., Haaker J., Heitland I., Hermann A., Kuhn M., Kruse O. (2017). Don’t fear ‘fear conditioning’: Methodological considerations for the design and analysis of studies on human fear acquisition, extinction, and return of fear. Neurosci. Biobehav. Rev..

[58] Duits P., Cath D.C., Lissek S., Hox J.J., Hamm A.O., Engelhard I.M., Van Den Hout M.A., Baas J.M.P. (2015). Updated meta-analysis of classical fear conditioning in the anxiety disorders. Depress. Anxiety.

[59] Lissek S., Powers A.S., McClure E.B., Phelps E.A., Woldehawariat G., Grillon C., Pine D.S. (2005). Classical fear conditioning in the anxiety disorders: A meta-analysis. Behav. Res. Ther..

[60] Kida S. (2019). Reconsolidation/destabilization, extinction and forgetting of fear memory as therapeutic targets for PTSD. Psychopharmacology.

[61] Bienvenu T.C.M., Dejean C., Jercog D., Aouizerate B., Lemoine M., Herry C. (2021). The advent of fear conditioning as an animal model of post-traumatic stress disorder: Learning from the past to shape the future of PTSD research. Neuron.

[62] Battaglia S., Di Fazio C., Vicario C.M., Avenanti A. (2023). Neuropharmacological modulation of N-methyl-D-aspartate, noradrenaline and endocannabinoid receptors in fear extinction learning: Synaptic transmission and plasticity. Int. J. Mol. Sci..

[63] Battaglia M.R., Di Fazio C., Battaglia S. (2023). Activated Tryptophan-Kynurenine Metabolic System in the Human Brain is Associated with Learned Fear. Front. Mol. Neurosci..

[64] Vicario C.M., Makris S., Culicetto L., Lucifora C., Falzone A., Martino G., Ferraioli F., Nitsche M.A., Avenanti A., Craparo G.C. (2023). Evidence of altered fear extinction learning in individuals with high vaccine hesitancy during COVID-19 pandemic. Clin. Neuropsychiatry.

[65] Maren S. (2001). Neurobiology of Pavlovian fear conditioning. Annu. Rev. Neurosci..

[66] Battaglia S., Thayer J.F. (2022). Functional interplay between central and autonomic nervous systems in human fear conditioning. Trends Neurosci..

[67] Haaker J., Maren S., Andreatta M., Merz C.J., Richter J., Richter S.H., Meir Drexler S., Lange M.D., Jüngling K., Nees F. (2019). Making translation work: Harmonizing cross-species methodology in the behavioural neuroscience of Pavlovian fear conditioning. Neurosci. Biobehav. Rev..

[68] Vicario C.M., Martino G. (2020). Dopamine and serotonin in fear extinction: Some key questions to be addressed. AIMS Neurosci..

[69] Zabik N.L., Peters C., Iadipaolo A., Marusak H.A., Rabinak C.A. (2023). Comparison of behavioral and brain indices of fear renewal during a standard vs. novel immersive reality Pavlovian fear extinction paradigm in healthy adults. Behav. Brain Res..

[70] Lucifora C., Grasso G.M., Perconti P., Plebe A. (2020). Moral dilemmas in self-driving cars. Riv. Internaz. Filos. Psicol..

[71] Lucifora C., Angelini L., Meteier Q., Vicario C.M., Khaled O.A., Mugellini E., Grasso G.M. (2021). Cyber-therapy: The use of artificial intelligence in psychological practice. Proceedings of the 4th International Conference on Intelligent Human Systems Integration (IHSI 2021): Integrating People and Intelligent Systems.

[72] Grasso G.M., Lucifora C., Perconti P., Plebe A. (2020). Integrating human acceptable morality in autonomous vehicles. Proceedings of the 3rd International Conference on Intelligent Human Systems Integration (IHSI 2020): Integrating People and Intelligent Systems.

[73] Grasso G.M., Lucifora C., Perconti P., Plebe A. Evaluating Mentalization during Driving. Proceedings of the 5th International Conference on Vehicle Technology and Intelligent Transport Systems (VEHITS 2019).

[74] Daher K., Capallera M., Lucifora C., Casas J., Meteier Q., El Kamali M., El Ali A., Grosso G.M., Chollet G., Abou Khaled O. Empathic interactions in automated vehicles# EmpathicCHI. Proceedings of the Extended Abstracts of the 2021 CHI Conference on Human Factors in Computing Systems.

[75] Lucifora C., Grasso G.M., Perconti P., Plebe A. (2020). Moral reasoning and automatic risk reaction during driving. Cogn. Technol. Work.

[76] Battaglia S., Garofalo S., di Pellegrino G., Starita F. (2020). Revaluing the role of vmPFC in the acquisition of pavlovian threat conditioning in humans. J. Neurosci..

[77] Fullana M.A., Dunsmoor J.E., Schruers K.R.J., Savage H.S., Bach D.R., Harrison B.J. (2020). Human fear conditioning: From neuroscience to the clinic. Behav. Res. Ther..

[78] Suarez-Jimenez B., Albajes-Eizagirre A., Lazarov A., Zhu X., Harrison B.J., Radua J., Neria Y., Fullana M.A. (2020). Neural signatures of conditioning, extinction learning, and extinction recall in posttraumatic stress disorder: A meta-analysis of functional magnetic resonance imaging studies. Psychol. Med..

[79] Vervliet B., Boddez Y. (2020). Memories of 100 years of human fear conditioning research and expectations for its future 2020. Behav. Res. Ther..

[80] Battaglia S., Orsolini S., Borgomaneri S., Barbieri R., Diciotti S., di Pellegrino G. (2022). Characterizing cardiac autonomic dynamics of fear learning in humans. Psychophysiology.

[81] Sehlmeyer C., Schöning S., Zwitserlood P., Pfleiderer B., Kircher T., Arolt V., Konrad C. (2009). Human fear conditioning and extinction in neuroimaging: A systematic review. PLoS ONE.

[82] Delgado M.R., Nearing K.I., LeDoux J.E., Phelps E.A. (2008). Neural Circuitry Underlying the Regulation of Conditioned Fear and Its Relation to Extinction. Neuron.

[83] LeDoux J. (1998). Fear and the brain: Where have we been, and where are we going?. Biol. Psychiatry.

[84] Corcoran K.A., Quirk G.J. (2007). Recalling Safety: Cooperative Functions of the Ventromedial Prefrontal Cortex and the Hippocampus in Extinction. CNS Spectr..

[85] Etkin A., Egner T., Kalisch R. (2011). Emotional processing in anterior cingulate and medial prefrontal cortex. Trends Cogn. Sci..

[86] Marković V., Vicario C.M., Yavari F., Salehinejad M.A., Nitsche M.A. (2021). A systematic review on the effect of transcranial direct current and magnetic stimulation on fear memory and extinction. Front. Hum. Neurosci..

[87] Quirk G.J., Mueller D. (2008). Neural mechanisms of extinction learning and retrieval. Neuropsychopharmacology.

[88] Vicario C.M., Nitsche M.A., Hoysted I., Yavari F., Avenanti A., Salehinejad M.A., Felmingham K.L. (2019). Anodal transcranial direct current stimulation over the ventromedial prefrontal cortex enhances fear extinction in healthy humans: A single blind sham-controlled study. Brain Stimul..

[89] Milad M.R., Quirk G.J., Pitman R.K., Orr S.P., Fischl B., Rauch S.L. (2007). A role for the human dorsal anterior cingulate cortex in fear expression. Biol. Psychiatry.

[90] Dixsaut L., Gräff J. (2021). The Medial Prefrontal Cortex and Fear Memory: Dynamics, Connectivity, and Engrams. Int. J. Mol. Sci..

[91] Johansen J.P., Hamanaka H., Monfils M.H., Behnia R., Deisseroth K., Blair H.T., LeDoux J.E. (2010). Optical activation of lateral amygdala pyramidal cells instructs associative fear learning. Proc. Natl. Acad. Sci. USA.

[92] Tye K.M., Prakash R., Kim S.-Y.Y., Fenno L.E., Grosenick L., Zarabi H., Thompson K.R., Gradinaru V., Ramakrishnan C., Deisseroth K. (2011). Amygdala circuitry mediating reversible and bidirectional control of anxiety. Nature.

[93] Rogan M.T., Stäubli U.V., LeDoux J.E. (1997). Fear conditioning induces associative long-term potentiation in the amygdala. Nature.

[94] Bouton M.E. (2004). Context and behavioral processes in extinction. Learn. Mem..

[95] Battaglia S., Cardellicchio P., Di Fazio C., Nazzi C., Fracasso A., Borgomaneri S. (2022). Stopping in (e) motion: Reactive action inhibition when facing valence-independent emotional stimuli. Front. Behav. Neurosci..

[96] Battaglia S., Cardellicchio P., Di Fazio C., Nazzi C., Fracasso A., Borgomaneri S. (2022). The influence of vicarious fear-learning in “infecting” reactive action inhibition. Front. Behav. Neurosci..

[97] Tovote P., Fadok J.P., Lüthi A. (2015). Neuronal circuits for fear and anxiety. Nat. Rev. Neurosci..

[98] Quirk G.J., Russo G.K., Barron J.L., Lebron K. (2000). The role of ventromedial prefrontal cortex in the recovery of extinguished fear. J. Neurosci..

[99] Milad M.R., Wright C.I., Orr S.P., Pitman R.K., Quirk G.J., Rauch S.L. (2007). Recall of fear extinction in humans activates the ventromedial prefrontal cortex and hippocampus in concert. Biol. Psychiatry.

[100] Barnes N.M., Sharp T. (1999). A review of central 5-HT receptors and their function. Neuropharmacology.

[101] Cornea-Hébert V., Riad M., Wu C., Singh S.K., Descarries L. (1999). Cellular and subcellular distribution of the serotonin 5-HT2A receptor in the central nervous system of adult rat. J. Comp. Neurol..

[102] Mascagni F., McDonald A.J. (2007). A novel subpopulation of 5-HT type 3A receptor subunit immunoreactive interneurons in the rat basolateral amygdala. Neuroscience.

[103] Rainnie D.G. (1999). Serotonergic modulation of neurotransmission in the rat basolateral amygdala. J. Neurophysiol..

[104] Bocchio M., McHugh S.B., Bannerman D.M., Sharp T., Capogna M. (2016). Serotonin, amygdala and fear: Assembling the puzzle. Front. Neural Circuits.

[105] Parent A., Descarries L., Beaudet A. (1981). Organization of ascending serotonin systems in the adult rat brain. A radioautographic study after intraventricular administration of [3H] 5-hydroxytryptamine. Neuroscience.

[106] Kawahara H., Yoshida M., Yokoo H., Nishi M., Tanaka M. (1993). Psychological stress increases serotonin release in the rat amygdala and prefrontal cortex assessed by in vivo microdialysis. Neurosci. Lett..

[107] Yokoyama M., Suzuki E., Sato T., Maruta S., Watanabe S., Miyaoka H. (2005). Amygdalic levels of dopamine and serotonin rise upon exposure to conditioned fear stress without elevation of glutamate. Neurosci. Lett..

[108] Mo B., Feng N., Renner K., Forster G. (2008). Restraint stress increases serotonin release in the central nucleus of the amygdala via activation of corticotropin-releasing factor receptors. Brain Res. Bull..

[109] Hashimoto S., Inoue T., Koyama T. (1999). Effects of conditioned fear stress on serotonin neurotransmission and freezing behavior in rats. Eur. J. Pharmacol..

[110] Yoshioka M., Matsumoto M., Togashi H., Saito H. (1995). Effects of conditioned fear stress on 5-HT release in the rat prefrontal cortex. Pharmacol. Biochem. Behav..

[111] McGonigle P., Ruggeri B. (2014). Animal models of human disease: Challenges in enabling translation. Biochem. Pharmacol..

[112] Bezchlibnyk-Butler K., Aleksic I., Kennedy S.H. (2000). Citalopram—A review of pharmacological and clinical effects. J. Psychiatry Neurosci..

[113] Gorman J.M. (2003). Treating generalized anxiety disorder. J. Clin. Psychiatry.

[114] Kent J.M., Coplan J.D., Gorman J.M. (1998). Clinical utility of the selective serotonin reuptake inhibitors in the spectrum of anxiety. Biol. Psychiatry.

[115] Klumpp H., Fitzgerald D.A., Cook E., Shankman S.A., Angstadt M., Phan K.L. (2014). Serotonin transporter gene alters insula activity to threat in social anxiety disorder. Neuroreport.

[116] Sheehan D.V., Raj B.A., Trehan R.R., Knapp E.L. (1993). Serotonin in panic disorder and social phobia. Int. Clin. Psychopharmacol..

[117] Moher D., Liberati A., Tetzlaff J., Altman D.G., Altman D., Antes G., Atkins D., Barbour V., Barrowman N., Berlin J.A. (2009). Preferred reporting items for systematic reviews and meta-analyses: The PRISMA statement. PLoS Med..

[118] Hensman R., Guimarães F.S., Wang M., Deakin J.F.W. (1991). Effects of ritanserin on aversive classical conditioning in humans. Psychopharmacology.

[119] Hetem L.A.B., De Souza C.J., Guimaràes F.S., Zuardi A.W., Graeff F.G. (1996). Effect of d-fenfluramine on human experimental anxiety. Psychopharmacology.

[120] Hellewell J.S.E., Guimaraes F.S., Wang M., Deakin J.F.W. (1999). Comparison of buspirone with diazepam and fluvoxamine on aversive classical conditioning in humans. J. Psychopharmacol..

[121] Silva M., Hetem L.A.B., Guimarães F.S., Graeff F.G. (2001). Opposite effects of nefazodone in two human models of anxiety. Psychopharmacology.

[122] Grillon C., Levenson J., Pine D.S. (2007). A single dose of the selective serotonin reuptake inhibitor citalopram exacerbates anxiety in humans: A fear-potentiated startle study. Neuropsychopharmacology.

[123] Grillon C., Chavis C., Covington M.F., Pine D.S. (2009). Two-week treatment with the selective serotonin reuptake inhibitor citalopram reduces contextual anxiety but not cued fear in healthy volunteers: A fear-potentiated startle study. Neuropsychopharmacology.

[124] Bui E., Orr S.P., Jacoby R.J., Keshaviah A., LeBlanc N.J., Milad M.R., Pollack M.H., Simon N.M. (2013). Two weeks of pretreatment with escitalopram facilitates extinction learning in healthy individuals. Hum. Psychopharmacol. Clin. Exp..

[125] Åhs F., Frick A., Furmark T., Fredrikson M. (2015). Human serotonin transporter availability predicts fear conditioning. Int. J. Psychophysiol..

[126] Lundquist P., Wilking H., Höglund A.U., Sandell J., Bergström M., Hartvig P., Långström B. (2005). Potential of [11C] DASB for measuring endogenous serotonin with PET: Binding studies. Nucl. Med. Biol..

[127] Hindi Attar C., Finckh B., Büchel C. (2012). The influence of serotonin on fear learning. PLoS ONE.

[128] Gorka S.M., Lieberman L., Klumpp H., Kinney K.L., Kennedy A.E., Ajilore O., Francis J., Duffecy J., Craske M.G., Nathan J. (2017). Reactivity to unpredictable threat as a treatment target for fear-based anxiety disorders. Psychol. Med..

[129] Kałużna-Czaplińska J., Gątarek P., Chirumbolo S., Chartrand M.S., Bjørklund G. (2019). How important is tryptophan in human health?. Crit. Rev. Food Sci. Nutr..

[130] Young S.N. (2013). Acute tryptophan depletion in humans: A review of theoretical, practical and ethical aspects. J. Psychiatry Neurosci. JPN.

[131] Robinson O.J., Overstreet C., Allen P.S., Pine D.S., Grillon C. (2012). Acute tryptophan depletion increases translational indices of anxiety but not fear: Serotonergic modulation of the bed nucleus of the stria terminalis?. Neuropsychopharmacology.

[132] Kanen J.W., Arntz F.E., Yellowlees R., Christmas D.M., Price A., Apergis-Schoute A.M., Sahakian B.J., Cardinal R.N., Robbins T.W. (2021). Effect of tryptophandepletion on conditioned threat memory expression: Role of intolerance of uncertainty. Biol. Psychiatry Cogn. Neurosci. Neuroimaging.

[133] Garpenstrand H., Annas P., Ekblom J., Oreland L., Fredrikson M. (2001). Human fear conditioning is related to dopaminergic and serotonergic biological markers. Behav. Neurosci..

[134] Lonsdorf T.B., Weike A.I., Nikamo P., Schalling M., Hamm A.O., Öhman A. (2009). Genetic gating of human fear learning and extinction: Possible implications for gene-environment interaction in anxiety disorder. Psychol. Sci..

[135] Crişan L.G., Panǎ S., Vulturar R., Heilman R.M., Szekely R., Drugǎ B., Dragoş N., Miu A.C. (2009). Genetic contributions of the serotonin transporter to social learning of fear and economic decision making. Soc. Cogn. Affect. Neurosci..

[136] Klumpers F., Heitland I., Oosting R.S., Kenemans J.L., Baas J.M.P. (2012). Genetic variation in serotonin transporter function affects human fear expression indexed by fear-potentiated startle. Biol. Psychol..

[137] Agren T., Furmark T., Eriksson E., Fredrikson M. (2012). Human fear reconsolidation and allelic differences in serotonergic and dopaminergic genes. Transl. Psychiatry.

[138] Agren T. (2014). Human reconsolidation: A reactivation and update. Brain Res. Bull..

[139] Hermann A., Küpper Y., Schmitz A., Walter B., Vaitl D., Hennig J., Stark R., Tabbert K. (2012). Functional Gene Polymorphisms in the Serotonin System and Traumatic Life Events Modulate the Neural Basis of Fear Acquisition and Extinction. PLoS ONE.

[140] Klucken T., Alexander N., Schweckendiek J., Merz C.J., Kagerer S., Osinsky R., Walter B., Vaitl D., Hennig J., Stark R. (2013). Individual differences in neural correlates of fear conditioning as a function of 5-HTTLPR and stressful life events. Soc. Cogn. Affect. Neurosci..

[141] Glotzbach-Schoon E., Andreatta M., Reif A., Ewald H., Tröger C., Baumann C., Deckert J., Mühlberger A., Pauli P. (2013). Contextual fear conditioning in virtual reality is affected by 5HTTLPR and NPSR1 polymorphisms: Effects on fear-potentiated startle. Front. Behav. Neurosci..

[142] Heitland I., Groenink L., Bijlsma E.Y., Oosting R.S., Baas J.M.P. (2013). Human Fear Acquisition Deficits in Relation to Genetic Variants of the Corticotropin Releasing Hormone Receptor 1 and the Serotonin Transporter. PLoS ONE.

[143] Heitland I., Groenink L., van Gool J.M., Domschke K., Reif A., Baas J.M.P. (2016). Human fear acquisition deficits in relation to genetic variants of the corticotropin-releasing hormone receptor 1 and the serotonin transporter—Revisited. Genes Brain Behav..

[144] Straube B., Reif A., Richter J., Lueken U., Weber H., Arolt V., Jansen A., Zwanzger P., Domschke K., Pauli P. (2014). The functional-1019C/G HTR1A polymorphism and mechanisms of fear. Transl. Psychiatry.

[145] Klumpers F., Kroes M.C., Heitland I., Everaerd D., Akkermans S.E.A., Oosting R.S., Van Wingen G., Franke B., Kenemans J.L., Fernández G. (2015). Dorsomedial prefrontal cortex mediates the impact of serotonin transporter linked polymorphic region genotype on anticipatory threat reactions. Biol. Psychiatry.

[146] Baas J.M.P.P., Heitland I. (2015). The impact of cue learning, trait anxiety and genetic variation in the serotonin 1A receptor on contextual fear. Int. J. Psychophysiol..

[147] Wannemüller A., Moser D., Kumsta R., Jöhren H.P., Margraf J. (2018). The Return of Fear: Variation of the Serotonin Transporter Gene Predicts Outcome of a Highly Standardized Exposure-Based One-Session Fear Treatment. Psychother. Psychosom..

[148] Schipper P., Hiemstra M., Bosch K., Nieuwenhuis D., Adinolfi A., Glotzbach S., Borghans B., Lopresto D., Fernández G., Klumpers F. (2019). The association between serotonin transporter availability and the neural correlates of fear bradycardia. Proc. Natl. Acad. Sci. USA.

[149] Sanders M.J., Wiltgen B.J., Fanselow M.S. (2003). The place of the hippocampus in fear conditioning. Eur. J. Pharmacol..

[150] Fullana M.A., Albajes-Eizagirre A., Soriano-Mas C., Vervliet B., Cardoner N., Benet O., Radua J., Harrison B.J. (2018). Fear extinction in the human brain: A meta-analysis of fMRI studies in healthy participants. Neurosci. Biobehav. Rev..

[151] Ney L.J., Vicario C.M., Nitsche M.A., Felmingham K.L. (2021). Timing matters: Transcranial direct current stimulation after extinction learning impairs subsequent fear extinction retention. Neurobiol. Learn. Mem..

[152] Van’t Wout M., Mariano T.Y., Garnaat S.L., Reddy M.K., Rasmussen S.A., Greenberg B.D. (2016). Can Transcranial Direct Current Stimulation Augment Extinction of Conditioned Fear?. Brain Stimul..

[153] Milad M.R., Quirk G.J. (2002). Neurons in medial prefrontal cortex signal memory for fear extinction. Nature.

[154] Sengupta A., Holmes A. (2019). A discrete dorsal raphe to basal amygdala 5-HT circuit calibrates aversive memory. Neuron.

[155] Abela A.R., Browne C.J., Sargin D., Prevot T.D., Ji X.D., Li Z., Lambe E.K., Fletcher P.J. (2020). Median raphe serotonin neurons promote anxiety-like behavior via inputs to the dorsal hippocampus. Neuropharmacology.

[156] Blakely R.D., Berson H.E., Fremeau R.T., Caron M.G., Peek M.M., Prince H.K., Bradley C.C. (1991). Cloning and expression of a functional serotonin transporter from rat brain. Nature.

[157] Lesch K.-P., Bengel D., Heils A., Sabol S.Z., Greenberg B.D., Petri S., Benjamin J., Müller C.R., Hamer D.H., Murphy D.L. (1996). Association of anxiety-related traits with a polymorphism in the serotonin transporter gene regulatory region. Science.

[158] Jakubovski E., Johnson J.A., Nasir M., Müller-Vahl K., Bloch M.H. (2019). Systematic review and meta-analysis: Dose–response curve of SSRIs and SNRIs in anxiety disorders. Depress. Anxiety.

[159] Edwards J.G., Anderson I. (1999). Systematic review and guide to selection of selective serotonin reuptake inhibitors. Drugs.

[160] Frazer A. (1997). Pharmacology of antidepressants. J. Clin. Psychopharmacol..

[161] Hiemke C., Härtter S. (2000). Pharmacokinetics of selective serotonin reuptake inhibitors. Pharmacol. Ther..

[162] Attenburrow M.-J., Mitter P., Whale R., Terao T., Cowen P. (2001). Low-dose citalopram as a 5-HT neuroendocrine probe. Psychopharmacology.

[163] Schulkin J., Morgan M.A., Rosen J.B. (2005). A neuroendocrine mechanism for sustaining fear. Trends Neurosci..

[164] Crockett M.J., Clark L., Roiser J.P., Robinson O.J., Cools R., Chase H.W., Den Ouden H., Apergis-Schoute A., Campbell-Meikeljohn D., Seymour B. (2012). Converging evidence for central 5-HT effects in acute tryptophan depletion. Mol. Psychiatry.

[165] Nishizawa S., Benkelfat C., Young S.N., Leyton M., de Mzengeza S., De Montigny C., Blier P., Diksic M. (1997). Differences between males and females in rates of serotonin synthesis in human brain. Proc. Natl. Acad. Sci. USA.

[166] Bel N., Artigas F. (1996). Reduction of serotonergic function in rat brain by tryptophan depletion: Effects in control and fluvoxamine-treated rats. J. Neurochem..

[167] Hettema J.M., Annas P., Neale M.C., Kendler K.S., Fredrikson M. (2003). A twin study of the genetics of fear conditioning. Arch. Gen. Psychiatry.

[168] Gordon J.A., Hen R. (2004). Genetic approaches to the study of anxiety. Annu. Rev. Neurosci..

[169] Dougherty L.R., Tolep M.R., Bufferd S.J., Olino T.M., Dyson M., Traditi J., Rose S., Carlson G.A., Klein D.N. (2013). Preschool anxiety disorders: Comprehensive assessment of clinical, demographic, temperamental, familial, and life stress correlates. J. Clin. Child Adolesc. Psychol..

[170] Watanabe A., Nakao K., Tokuyama M., Takeda M. (2005). Prediction of first episode of panic attack among white-collar workers. Psychiatry Clin. Neurosci..

[171] Nugent N.R., Tyrka A.R., Carpenter L.L., Price L.H. (2011). Gene–environment interactions: Early life stress and risk for depressive and anxiety disorders. Psychopharmacology.

[172] Lonsdorf T.B., Kalisch R. (2011). A review on experimental and clinical genetic associations studies on fear conditioning, extinction and cognitive-behavioral treatment. Transl. Psychiatry.

[173] Hariri A.R., Mattay V.S., Tessitore A., Kolachana B., Fera F., Goldman D., Egan M.F., Weinberger D.R. (2002). Serotonin transporter genetic variation and the response of the human amygdala. Science.

[174] Canli T., Lesch K.-P. (2007). Long story short: The serotonin transporter in emotion regulation and social cognition. Nat. Neurosci..

[175] Dannlowski U., Konrad C., Kugel H., Zwitserlood P., Domschke K., Schöning S., Ohrmann P., Bauer J., Pyka M., Hohoff C. (2010). Emotion specific modulation of automatic amygdala responses by 5-HTTLPR genotype. Neuroimage.

[176] Heinz A., Braus D.F., Smolka M.N., Wrase J., Puls I., Hermann D., Klein S., Grüsser S.M., Flor H., Schumann G. (2005). Amygdala-prefrontal coupling depends on a genetic variation of the serotonin transporter. Nat. Neurosci..

[177] Munafo M.R., Clark T., Flint J. (2005). Does measurement instrument moderate the association between the serotonin transporter gene and anxiety-related personality traits? A meta-analysis. Mol. Psychiatry.

[178] Ippolito G., Bertaccini R., Tarasi L., Di Gregorio F., Trajkovic J., Battaglia S., Romei V. (2022). The role of alpha oscillations among the main neuropsychiatric disorders in the adult and developing human brain: Evidence from the last 10 years of research. Biomedicines.

[179] Di Gregorio F., La Porta F., Petrone V., Battaglia S., Orlandi S., Ippolito G., Romei V., Piperno R., Lullini G. (2022). Accuracy of EEG biomarkers in the detection of clinical outcome in disorders of consciousness after severe acquired brain injury: Preliminary results of a pilot study using a machine learning approach. Biomedicines.

[180] Tanaka M., Diano M., Battaglia S. (2023). Editorial: Insights into structural and functional organization of the brain: Evidence from neuroimaging and non-invasive brain stimulation techniques. Front. Psychiatry.

[181] Di Gregorio F., Battaglia S. (2023). Advances in EEG-based functional connectivity approaches to the study of the central nervous system in health and disease. Adv. Clin. Exp. Med..

[182] Bennett A.J., Lesch K.P., Heils A., Long J.C., Lorenz J.G., Shoaf S.E., Champoux M., Suomi S.J., Linnoila M.V., Higley J.D. (2002). Early experience and serotonin transporter gene variation interact to influence primate CNS function. Mol. Psychiatry.

[183] Songtachalert T., Roomruangwong C., Carvalho A.F., Bourin M., Maes M. (2018). Anxiety Disorders: Sex Differences in Serotonin and Tryptophan Metabolism. Curr. Top. Med. Chem..

[184] Mitsushima D., Yamada K., Takase K., Funabashi T., Kimura F. (2006). Sex differences in the basolateral amygdala: The extracellular levels of serotonin and dopamine, and their responses to restraint stress in rats. Eur. J. Neurosci..

[185] Willadsen M., Üngör M., Sługocka A., Schwarting R.K.W., Homberg J.R., Wöhr M. (2021). Fear Extinction and Predictive Trait-Like Inter-Individual Differences in Rats Lacking the Serotonin Transporter. Int. J. Mol. Sci..

[186] Barton C., Sklenicka J., Sayegh P., Yaffe K., Schubert C.C., Boustani M., Callahan C.M., Perkins A.J., Carney C.P., Fox C. (2003). A double-blind trial of bupropion versus desipramine for bipolar depression. J. Clin. Psychiatry.

[187] Pettersson R., Hagsäter S.M., Eriksson E. (2016). Serotonin depletion eliminates sex differences with respect to context-conditioned immobility in rat. Psychopharmacology.

[188] Donner N.C., Lowry C.A. (2013). Sex differences in anxiety and emotional behavior. Pflugers Arch..

[189] Vicario C.M., Nitsche M.A., Felmingham K. (2017). Forgetting fear associations through tES: Which memory process might be critical?. Transl. Psychiatry.

[190] Rubio-Godoy M., Aunger R., Curtis V. (2007). Serotonin—A link between disgust and immunity?. Med. Hypotheses.

[191] Limebeer C.L., Parker L.A., Fletcher P.J. (2004). 5,7-dihydroxytryptamine lesions of the dorsal and median raphe nuclei interfere with lithium-induced conditioned gaping, but not conditioned taste avoidance, in rats. Behav. Neurosci..

[192] Vicario C.M. (2013). Altered insula response to sweet taste processing in recovered anorexia and bulimia nervosa: A matter of disgust sensitivity?. Am. J. Psychiatry.

[193] Vicario C.M., Rafal R.D., Borgomaneri S., Paracampo R., Kritikos A., Avenanti A. (2017). Pictures of disgusting foods and disgusted facial expressions suppress the tongue motor cortex. Soc. Cogn. Affect. Neurosci..

[194] Vicario C.M., Rafal R.D., Di Pellegrino G., Lucifora C., Salehinejad M.A., Nitsche M.A., Avenanti A. (2022). Indignation for moral violations suppresses the tongue motor cortex: Preliminary TMS evidence. Soc. Cogn. Affect. Neurosci..

[195] Vicario C.M., Lucifora C. (2021). Neuroethics: What the study of brain disorders can tell about moral behavior. AIMS Neurosci..

[196] Lucifora C., Martino G., Curcuruto A., Salehinejad M.A., Vicario C.M. (2021). How self-control predicts moral decision making: An exploratory study on healthy participants. Int. J. Environ. Res. Public Health.

[197] Vicario C.M., Turrini S., Lucifora C., Culicetto L., Ferraioli F., Falzone A., Nitsche M.A., Avenanti A. (2022). When defeat leaves a bad taste in the mouth: Modulation of tongue corticobulbar output during monetary loss in a gambling task. Brain Stimul..

[198] Vicario C.M., Rafal R.D., Avenanti A. (2015). Counterfactual thinking affects the excitability of the motor cortex. Cortex.

[199] Coppini S., Lucifora C., Vicario C.M., Gangemi A. (2023). Experiments on real-life emotions challenge Ekman’s model. Sci. Rep..

[200] Barchetta S., Martino G., Craparo G., Salehinejad M.A., Nitsche M.A., Vicario C.M. (2021). Alexithymia is linked with a negative bias for past and current events in healthy humans. Int. J. Environ. Res. Public Health.

[201] Borgomaneri S., Vitale F., Avenanti A. (2015). Early changes in corticospinal excitability when seeing fearful body expressions. Sci. Rep..

[202] Borgomaneri S., Vitale F., Avenanti A. (2017). Behavioral inhibition system sensitivity enhances motor cortex suppression when watching fearful body expressions. Brain Struct. Funct..

[203] Andreatta M., Pauli P. (2021). Contextual modulation of conditioned responses in humans: A review on virtual reality studies. Clin. Psychol. Rev..

[204] Andreatta M., Winkler M.H., Collins P., Gromer D., Gall D., Pauli P., Gamer M., Geyer M.A., Marsden C.A., Ellenbroek B.A., Barnes T.R.E., Andersen S.L., Paulus M.P., Olivier J. (2023). VR for studying the neuroscience of emotional responses. Current Topics in Behavioral Neurosciences.

[205] Fiori F., Chiappini E., Soriano M., Paracampo R., Romei V., Borgomaneri S., Avenanti A. (2017). Long-latency interhemispheric interactions between motor-related areas and the primary motor cortex: A dual site TMS study. Sci. Rep..

[206] Fiori F., Chiappini E., Avenanti A. (2018). Enhanced action performance following TMS manipulation of associative plasticity in ventral premotor-motor pathway. Neuroimage.

[207] Chiappini E., Borgomaneri S., Marangon M., Turrini S., Romei V., Avenanti A. (2020). Driving associative plasticity in premotor-motor connections through a novel paired associative stimulation based on long-latency cortico-cortical interactions. Brain Stimul..

[208] Valchev N., Tidoni E., Hamilton A.F.D.C., Gazzola V., Avenanti A. (2017). Primary somatosensory cortex necessary for the perception of weight from other people’s action: A continuous theta-burst TMS experiment. Neuroimage.

[209] Turrini S., Fiori F., Chiappini E., Lucero B., Santarnecchi E., Avenanti A. (2023). Cortico-cortical paired associative stimulation (ccPAS) over premotor-motor areas affects local circuitries in the human motor cortex via Hebbian plasticity. Neuroimage.

[210] Lefaucheur J.P., Aleman A., Baeken C., Benninger D.H., Brunelin J., Di Lazzaro V., Filipović S.R., Grefkes C., Hasan A., Hummel F.C. (2020). Evidence-based guidelines on the therapeutic use of repetitive transcranial magnetic stimulation (rTMS): An update (2014–2018). Clin. Neurophysiol..

[211] Avenanti A., Coccia M., Ladavas E., Provinciali L., Ceravolo M.G. (2012). Low-frequency rTMS promotes use-dependent motor plasticity in chronic stroke: A randomized trial. Neurology.

[212] Salehinejad M.A., Nejati V., Mosayebi-Samani M., Mohammadi A., Wischnewski M., Kuo M.F., Avenanti A., Vicario C.M., Nitsche M.A. (2020). Transcranial direct current stimulation in ADHD: A systematic review of efficacy, safety, and protocol-induced electrical field modeling results. Neurosci. Bull..

[213] Casula A., Milazzo B.M., Martino G., Sergi A., Lucifora C., Tomaiuolo F., Quartarone A., Nitsche M.A., Vicario C.M. (2023). Non-Invasive brain stimulation for the modulation of aggressive behavior-A systematic review of randomized sham-controlled studies. Life.

[214] Turrini S., Wong B., Eldaief M., Press D.Z., Sinclair D.A., Koch G., Avenanti A., Santarnecchi E. (2023). The multifactorial nature of healthy brain ageing: Brain changes, functional decline and protective factors. Ageing Res. Rev..

[215] Vicario C.M., Salehinejad M.A., Felmingham K., Martino G., Nitsche M.A. (2019). A systematic review on the therapeutic effectiveness of non-invasive brain stimulation for the treatment of anxiety disorders. Neurosci. Biobehav. Rev..

[216] Vicario C.M., Salehinejad M.A., Avenanti A., Nitsche M.A., Dell’Osso B., Di Lorenzo G. (2020). Transcranial direct current stimulation (tDCS) in anxiety disorders. Non Invasive Brain Stimulation in Psychiatry and Clinical Neurosciences.

[217] Vicario C.M., Salehinejad M.A., Lucifora C., Martino G., Falzone A.M., Grasso G., Nitsche Michael A., Pinna G. (2023). Combining virtual reality exposure therapy with non-invasive brain stimulation for the treatment of post-traumatic stress disorder and related syndromes: A perspective. Translational Methods for PTSD Research.

